# Impaired Cytokine Secretion Contributes to Age‐Dependent Immune Dysfunction in SARS Coronavirus Response and Is Restored by Young CD11b‐Positive Cell Transfer

**DOI:** 10.1111/acel.70154

**Published:** 2025-06-29

**Authors:** Yu‐Xuan Wu, Chih‐Wei Hu, Jui‐Yu Chang, Shuoh‐Wen Chen, Ting‐An Lin, Eric Chang‐Yi Lin, Min‐Hui Chen, Chuen‐Mi Yang, Hsuan‐Ying Lu, Ching‐Jung Teng, Yuan‐I Chang

**Affiliations:** ^1^ Institute of Physiology, College of Medicine National Yang Ming Chiao Tung University Taipei City Taiwan; ^2^ Experimental Animal Center, Institute of Preventive Medicine National Defense Medical College New Taipei City Taiwan; ^3^ Department of Biotechnology and Laboratory Science in Medicine, College of Biomedical Science and Engineering National Yang Ming Chiao Tung University Taipei City Taiwan; ^4^ Department of Internal Medicine, College of Medicine National Yang Ming Chiao Tung University Taipei City Taiwan; ^5^ Division of Hematology, Department of Medicine Taipei Veterans General Hospital Taipei City Taiwan; ^6^ Department of Orthopedics and Rehabilitation University of Wisconsin‐Madison Madison Wisconsin USA; ^7^ School of Medicine, College of Medicine National Yang Ming Chiao Tung University Taipei City Taiwan

**Keywords:** aging, innate immunity, IRF7, SARS‐CoV, SNARE

## Abstract

COVID‐19 mortality disproportionately affects the elderly, yet the cellular and molecular factors contributing to age‐related immune system remodeling remain unclear. Using SARS‐CoV‐derived ssRNA sequences, we modeled age‐dependent immune responses in mice. Aged mice exhibited higher mortality and severe lung inflammation upon viral ssRNA challenge, mirroring clinical observations. We uncovered a pre‐existing inflammatory state in aged mice, characterized by elevated baseline levels of specific immune cells and cytokines correlating with poor outcomes. Age‐related immune dysfunction stemmed from impaired IRF7 signaling and defective SNARE‐mediated cytokine secretion in CD11b^+^ cells. Notably, the adoptive transfer of young CD11b^+^ cells to aged mice exposed to SARS‐CoV2 ssRNA reduced mortality, alleviated lung inflammation, and normalized cytokine profiles. These findings provide insights into age‐related immune dysregulation during viral challenges and suggest potential therapeutic strategies for severe COVID‐19 in the elderly.

## Graphical abstract

Aged CD11b^+^ cells exhibit impaired IRF7 activation and reduced SNARE protein expression, leading to delayed interferon responses and diminished cytokine secretion. These dual deficits may contribute to the heightened susceptibility of older individuals to severe outcomes following SARS‐CoV infection.

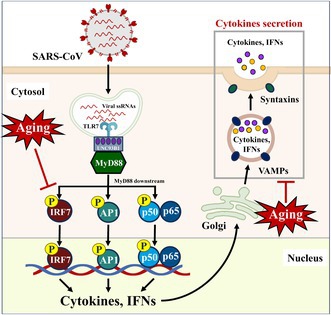

**Abbreviations**
ACE2: angiotensin‐converting enzyme 2
APC: antigen‐presenting cell
CBA: cytometric bead array
CDC: Centers for Disease Control and Prevention
COVID‐19: coronavirus disease of 2019
DOTAP: 1,2‐dioleoyl‐3‐trimethylammonium‐propane
HIV: human immunodeficiency virus
IFN: interferon
IRF7: interferon regulatory factor 7
MAPK: mitogen‐activated protein kinase
MFI: mean fluorescence intensities
MyD88: myeloid differentiation primary response 88
NF‐κB: nuclear factor kappa‐light‐chain‐enhancer of activated B cells
PAMP: pathogen‐associated molecular pattern
PFU: plaque‐forming units
PRR: pattern‐recognition receptor
RBD: receptor‐binding domain
S: spike protein
SARS‐CoV: severe acute respiratory syndrome coronavirus
SNARE: soluble N‐ethylmaleimide‐sensitive factor attachment protein receptor
ssRNA: single‐strand RNA
TLR: toll‐like receptor
WBC: white blood cell
WT: wild‐type

## 1 Introduction

The 21st century has witnessed global health emergencies caused by coronaviruses, with outbreaks of severe acute respiratory syndrome coronavirus 1 (SARS‐CoV‐1) in 2002 and SARS‐CoV‐2 in 2019 posing significant challenges (Peiris et al. 2003; Shereen et al. 2020). The coronavirus disease of 2019 (COVID‐19) pandemic, caused by SARS‐CoV‐2, has disproportionately impacted the elderly population, highlighting the critical role of age‐related immune dysfunction in disease severity (Cortis 2020; Romero Starke et al. 2020). As individuals age, both innate and adaptive immune responses decline, reducing antimicrobial capabilities and increasing vulnerability to infections. Additionally, inflammageing, characterized by a systemic state of chronic low‐grade inflammation, exacerbates disease progression and weakens vaccine efficacy in older adults (Lee et al. 2022). The Centers for Disease Control and Prevention (CDC) has recognized the heightened risk of COVID‐19 infection, hospitalization, and mortality with advancing age (Castilla et al. 2021). However, the specific mechanisms underlying the altered immune responses in elderly individuals infected with SARS‐CoV‐2 remain incompletely understood, hindering efforts to mitigate the impact of COVID‐19 on this vulnerable population.

In viral infections, the innate immune response plays a crucial role in recognizing pathogen‐associated molecular patterns (PAMPs) through pattern‐recognition receptors (PRRs), such as toll‐like receptors (TLRs) (Kaisho and Akira 2001). TLR7, found in antigen‐presenting cells (APCs) like dendritic cells (DCs), monocytes, and macrophages, is responsible for detecting single‐stranded RNA (ssRNA) viruses. Upon activation, TLR7 initiates the myeloid differentiation primary response 88 (MyD88) signaling cascade, involving downstream molecules like nuclear factor kappa‐light‐chain‐enhancer of activated B cells (NF‐κB), interferon regulatory factors (IRFs), and mitogen‐activated protein kinases (MAPKs). This cascade leads to the expression of pro‐inflammatory cytokines and type I interferons (IFNs), which are crucial for viral replication control (Diebold et al. 2004). Recent studies have highlighted the importance of SNARE (soluble N‐ethylmaleimide‐sensitive factor attachment protein receptor) proteins in cytokine secretion from immune cells (Murray and Stow 2014). These proteins mediate vesicle fusion and are essential for the release of cytokines and other immune mediators. However, the role of SNARE proteins in age‐related immune dysfunction, particularly in the context of viral infections, remains largely unexplored.

To study SARS‐CoV‐2 pathogenesis, researchers have faced challenges due to structural differences between human and mouse ACE2 receptors (Winkler et al. 2020; Yang et al. 2007). Although some have used pseudoviruses expressing human ACE2‐coding sequences to infect mice, this method still poses biosafety risks (Rawle et al. 2021). An alternative approach leverages GU‐rich ssRNA, such as human immunodeficiency virus (HIV)‐derived ssRNA40, to stimulate TLR7 and mimic viral infection in mouse models (Alter et al. 2007; Giraldo et al. 2016). Notably, previous studies have successfully employed SARS‐CoV‐derived ssRNAs to investigate immune responses in both mouse models and human cells (Li et al. 2013; Salvi et al. 2021). Building on these advances, we used SARS‐CoV‐related ssRNAs to investigate age‐associated changes in immune responses, elucidate underlying mechanisms, and explore potential therapeutic approaches.

In this study, we examined immune responses in young (8–12 weeks) and elderly (18–24 months) C57BL/6J mice treated with GU‐rich ssRNA fragments derived from SARS‐CoV‐1 and SARS‐CoV‐2. This approach provided a safer and more cost‐effective means of studying the pathophysiological processes associated with SARS‐CoV infection in vivo. We investigated the mechanisms underlying immune responses in the elderly, focusing on cytokine production and secretion following ssRNA stimulation. Additionally, we explored the potential of young immune cell transfer as a therapeutic strategy for enhancing immune responses in aged individuals. These findings may inform the development of age‐specific therapeutic strategies and interventions to combat SARS‐CoV‐2 infection and other viral diseases that disproportionately affect older populations.

## 2 Results

### 2.1 Identification and Functional Validation of Conserved GU‐Rich ssRNA Sequences From SARS‐CoV Genomes

**FIGURE 1:**
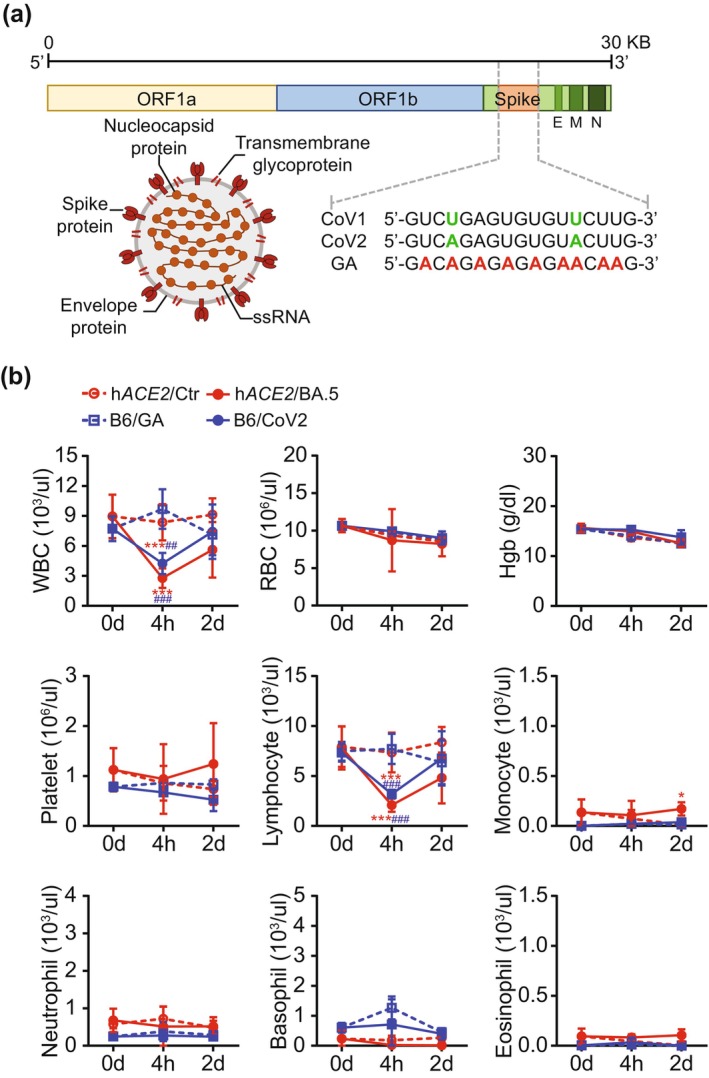
Comparison of SARS‐CoV‐derived GU‐rich ssRNA sequences and their hematological effects versus live SARS‐CoV‐2 BA.5 variant. (a) Schematic of the SARS‐CoV family genome, highlighting the location of identified GU‐rich ssRNA sequences. Sequences of CoV1 (SARS‐CoV‐1), CoV2 (SARS‐CoV‐2), and control GA ssRNAs are shown. (b) Hematological profiles in young mice following CoV2 ssRNA administration (*n* = 5) or live SARS‐CoV‐2 BA.5 variant infection (*n* = 4–6). Young C57BL/6 mice (B6, 8–12 weeks) received CoV2 ssRNA (1 mg/kg, retro‐orbital injection) or GA ssRNA (control). Age‐matched transgenic B6.129S2(Cg)‐*Ace2* ^*tm1(ACE2)Dwnt*^/J (hACE2) mice were infected intranasally with live SARS‐CoV‐2 BA.5 variant (5 × 10^4^ PFU) or DMEM (control). Blood samples were analyzed at baseline (0 h), 4 h, and 2 days post‐treatment. Parameters: White blood cells (WBC), red blood cells (RBC), hemoglobin (Hgb), platelets, lymphocytes, monocytes, neutrophils, basophils, and eosinophils. Data are presented as mean ± SD. Statistical significance was determined using two‐way ANOVA with Tukey's post hoc test. **p < 0.01, ***p < 0.001 compared to h*ACE2*/Ctr. ^##^ *p* < 0.01, ^###^ *p* < 0.001 compared to B6/GA.

We investigated immunostimulatory sequences within SARS‐CoV genomes, inspired by previous research on ssRNA40, a GU‐rich single‐stranded RNA from HIV‐1's U5 region that acts as a natural agonist of TLR7 (Alter et al. 2007; Giraldo et al. 2016). We screened for GU‐rich sequences with > 40% GU pair content and at least one “UGUGU” IFN induction motif  within ~20 bases (Lehnardt et al. 2019; Rolle et al. 2016). Focusing on the spike (S) gene region, we identified similar sequences in both SARS‐CoV‐1 (5′‐GUCUGAGUGUGUUCUUG‐3′) (Li et al. 2013) and the original SARS‐CoV‐2 strain (5′‐GUCAGAGUGUGUACUUG‐3′), differing by only two base pairs (Figure 1a). Analysis of all known SARS‐CoV‐2 variants confirmed these sequences are highly conserved (Table 1), suggesting their potential significance in viral pathogenesis across strains.

**TABLE 1:** Sequence alignment of GU‐rich ssRNA motifs from the genomic sequence of spike protein of SARS‐CoV‐2 original strain and various mutant strains.

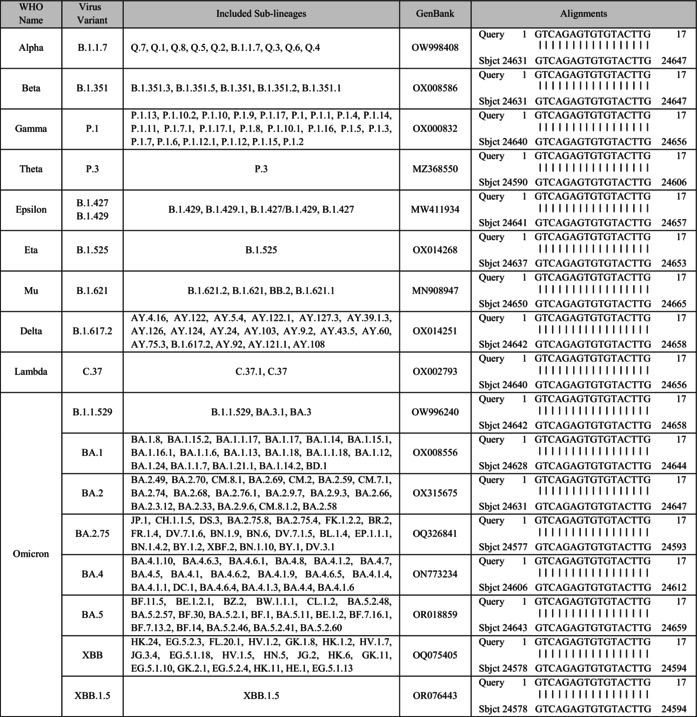

*Note:* This table presents the alignment results of GU‐rich ssRNA sequences. WHO Name: The World Health Organization (WHO) designated name of the variant. Virus Variant: The lineage designation of the virus variant. Included Sub‐lineages: Sub‐lineages included within each variant category. GenBank: The GenBank accession number of the reference genome for each variant. Alignments: The alignment results, where ‘Query’ represents the sequence from the original SARS‐CoV‐2 strain and ‘Sbjct’ represents the sequence from the respective mutant strain. The numerical values denote the position of the sequences within the viral genome, highlighting the highly conserved regions with only minimal base pair differences.

Clinical studies have demonstrated that plasma levels of SARS‐CoV‐2 RNA can be quantified using standardized RT‐PCR techniques, and SARS‐CoV‐2 RNAemia is correlated with disease severity, including increased cytokines and chemokines, C‐reactive protein, ferritin, coagulation activation, tissue damage, and extrapulmonary complications (Jacobs and Mellors 2021; Ram‐Mohan et al. 2022). To investigate the immunostimulatory potential of these conserved sequences, we synthesized ssRNAs corresponding to SARS‐CoV‐1 (CoV1), SARS‐CoV‐2 (CoV2), and a control ssRNA with U/A alternations (GA) (Li et al. 2013; Salvi et al. 2021) (Figure 1a). We validated the biological activity of our synthetic CoV2‐GU ssRNA by comparing its effects to those of live SARS‐CoV‐2 (BA.5 variant) infection in vivo. The BA.5 variant, characterized by S protein mutations L452R, F486V, and R493Q in the receptor‐binding domain (RBD), is known for enhanced viral attachment and immune evasion (Liu et al. 2024; Sabbatucci et al. 2023). We infected young transgenic mice expressing human ACE2 (hACE2) with live SARS‐CoV‐2 BA.5 virus (5 × 10^4^ PFU) or injected young wild‐type (WT) mice (C57BL/6J) with 1,2‐dioleoyl‐3‐trimethylammonium‐propane (DOTAP)‐encapsulated CoV2 ssRNA (1 mg/kg body weight) via the retro‐orbital sinus. Peripheral blood analysis at 0, 4 h, and 2 days post‐treatment revealed similar effects for both CoV2 ssRNA and live virus (Figure 1b). Both treatments led to significant decreases in white blood cell (WBC) and lymphocyte counts at 4 h post‐administration. WBC counts decreased by approximately 44% in CoV2 ssRNA‐treated mice and 33% in BA.5‐infected mice, while lymphocyte counts were reduced by approximately 41% and 28%, respectively, when compared to their corresponding control groups at 4 h. These parameters returned to pre‐treatment levels by day 2, suggesting a rapid but transient immune response in young mice (Figure 1b).

These findings demonstrate that our synthetic CoV2 ssRNA can elicit immune responses comparable to those induced by live SARS‐CoV‐2 virus. This parallel response not only validates our synthetic ssRNAs as a valuable research tool but also provides insights into the early immune dynamics of SARS‐CoV‐2 infection. In subsequent experiments, we utilized young (8–12 weeks) and aged (18–24 months) mice to explore these age‐related variations, aiming to unravel the mechanisms behind the increased COVID‐19 severity observed in older populations.

### 2.2 Age‐Dependent Mortality and Lung Inflammation in Mice Following SARS‐CoV ssRNAs Administration

Clinical reports indicate that ~80% of COVID‐19‐related deaths occur in people aged 65 and over, with severe lung infection and excessive inflammatory responses leading to organ failure as primary causes of mortality (Tisminetzky et al. 2022). We investigated whether viral GU‐rich ssRNAs could induce similar age‐dependent pathophysiological phenotypes and mortality in our mouse model.

**FIGURE 2:**
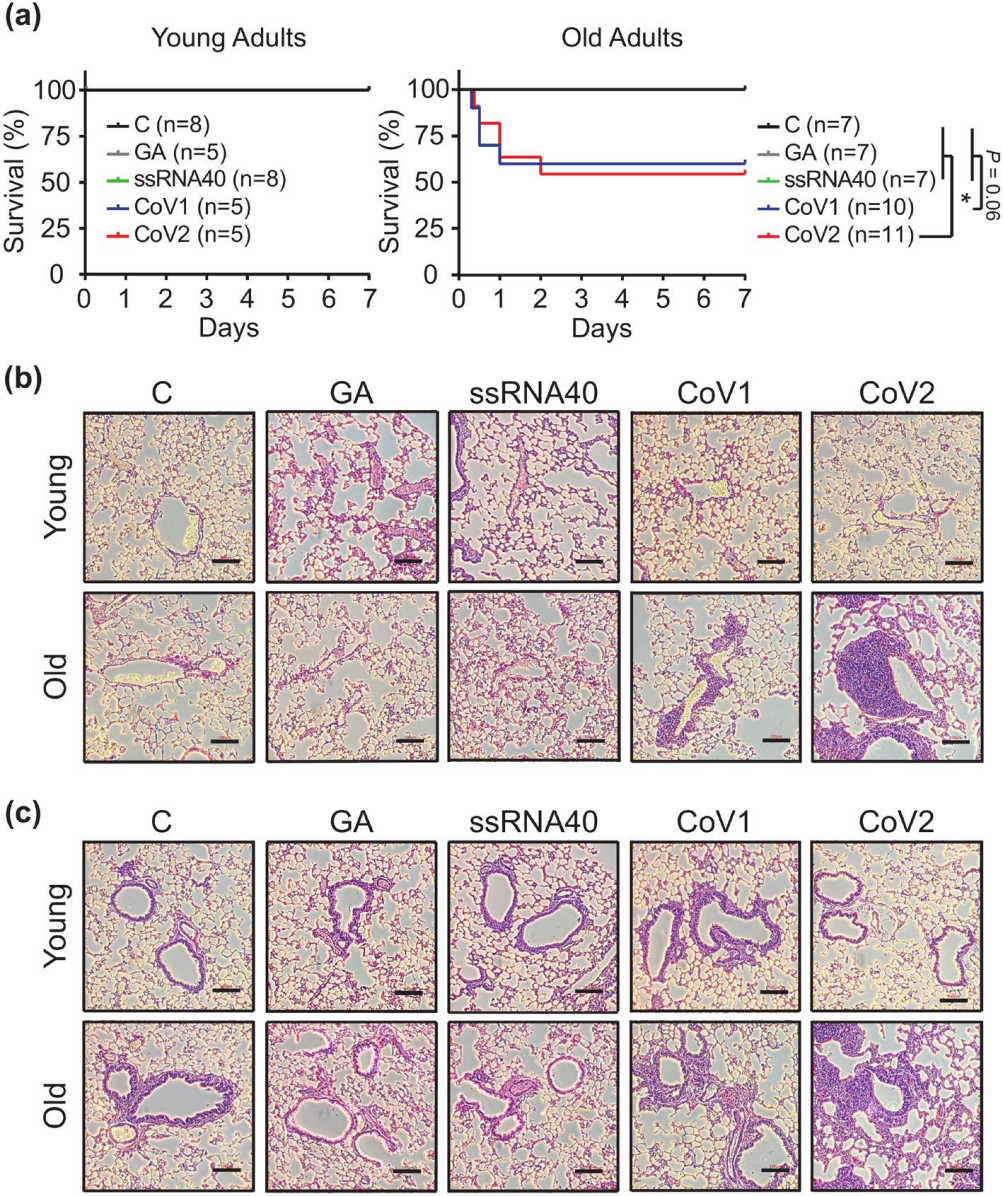
SARS‐CoV ssRNAs induce age‐dependent mortality and lung inflammation in mice. (a) Kaplan–Meier survival curves of young (8–12 weeks) and aged (18–24 months) mice following retro‐orbital injection of CoV1, CoV2, GA ssRNA, ssRNA40 (all at 1 mg/kg), or DOTAP reagent control (C). Statistical significance was determined using log‐rank Mantel‐Cox test. *p < 0.05. Representative hematoxylin and eosin (H&E) stained lung sections showing (b) veins and (c) bronchial areas from young and aged mice 7 days after injection with the indicated ssRNAs or control. Scale bar: 100 μm.

We administered SARS‐CoV ssRNAs via retro‐orbital injection into young and aged mice and observed severe outcomes, including weakness, lethargy, and mortality, specifically in the aged group. Survival analysis revealed a striking age‐dependent susceptibility to SARS‐CoV ssRNAs. Aged mice injected with CoV2 ssRNA showed significantly lower survival rates compared to control groups, including DOTAP reagent control, GA‐ and ssRNA40‐treated mice (54.55% vs. 100%, *p* < 0.05, log‐rank test). CoV1 ssRNA treatment in aged mice exhibited a similar trend towards decreased survival (60% survival, *p* = 0.06). All young mice maintained 100% survival across all treatment groups, underscoring the robust age‐related difference in SARS‐CoV ssRNA‐induced mortality (Figure 2a).

Histopathological analysis of lung tissues revealed significant immune cell infiltration surrounding veins and bronchial areas, primarily in aged mice treated with CoV1 and CoV2 ssRNAs. This infiltration was characterized by abundant leukocytes around lung veins (Figure 2b), with alveolar wall thickening and reduced airspace in bronchial areas (Figure 2c). The pattern of age‐dependent differential immune cell infiltration observed in lung tissues parallels that were seen in hACE2 transgenic mice infected with live SARS‐CoV‐2 virus (Figure S1). The DOTAP control, GA‐, and ssRNA40‐treated groups showed no significant infiltration in either age group (Figure 2b,c), exhibiting histopathological profiles comparable to those observed in young and aged hACE2 mice receiving intranasal DMEM medium administration as controls (Figure S1, Control groups). CoV1 and CoV2 ssRNA exposure did not induce histological changes in the spleens of either young or aged mice (Figure S2), highlighting tissue‐specific immune responsiveness likely shaped by local receptor expression and vascular accessibility (Bonam et al. 2024). These findings demonstrate that CoV1 and CoV2 ssRNAs induced higher mortality rates and acute pneumonia‐like symptoms in aged mice compared to young mice, partially recapitulating the increased COVID‐19 severity observed in elderly humans.

### 2.3 Temporal Immune Cell Changes and Pre‐Challenge Status Determine Age‐Specific Responses to SARS‐CoV ssRNAs

Aging profoundly impacts the immune system, affecting both innate and adaptive responses. Our study investigated the impact of aging on immune cell populations in C57BL/6 mice, aiming to firstly establish reference values for age‐related changes and identify potential strategies to mitigate severe COVID‐19 risks in aged individuals. We first analyzed baseline differences in immune cell compartments between young and aged mice through a complete blood count (CBC) test. Aged mice exhibited higher counts of white blood cells (WBCs), neutrophils, monocytes, lymphocytes, and platelets but showed signs of anemia (Figure S3a). Flow cytometric analysis of white blood cell subtype populations revealed higher frequencies of CD11b^+^ myeloid cells and B cells, while CD4^+^ T cell frequency was lower in aged mice (Figure S3b).

**FIGURE 3:**
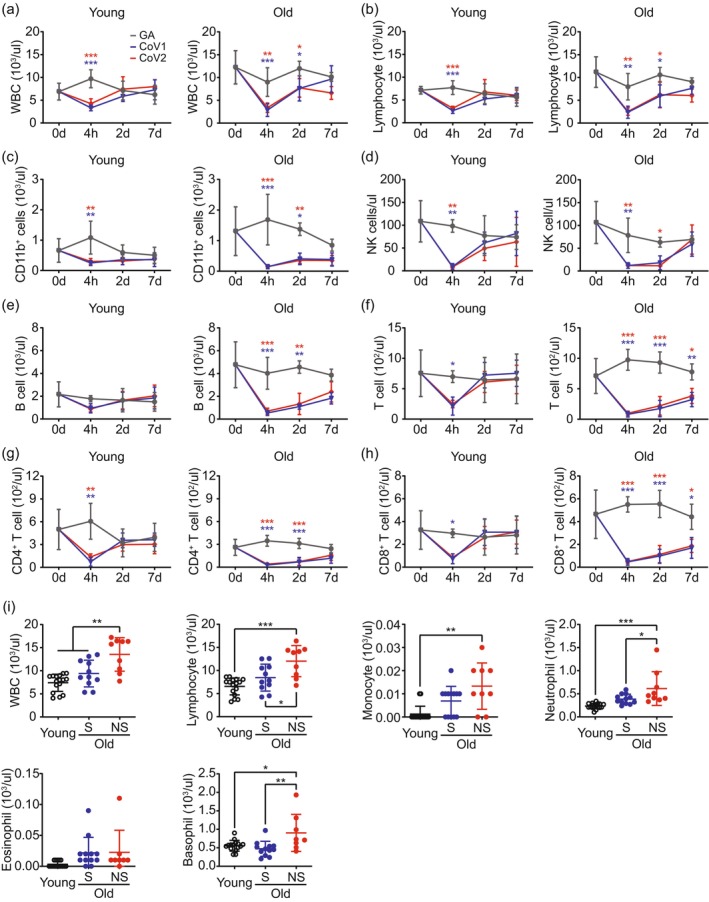
Age‐dependent immune cell dynamics and baseline hematological profiles correlating with survival outcomes. (a–h) Temporal analysis of peripheral blood immune cells in young and aged mice after injection with GA (gray), CoV1 (blue), or CoV2 (red) ssRNAs. Blood samples were collected at baseline (0‐h, *n* = 12–14), 4 h, 2 days, and 7 days post‐injection (*n* = 3–5). Analyzed parameters: (a) White blood cells (WBC); (b) Lymphocytes; (c) CD11b^+^ cells; (d) Natural killer (NK) cells; (e) B cells; (f) Total T cells; (g) CD4^+^ T cells; (h) CD8^+^ T cells. (i) Comparison of baseline hematological parameters among young mice, surviving (S) and non‐surviving (NS) aged mice following ssRNA injections. Parameters include WBC, lymphocytes, monocytes, neutrophils, eosinophils, and basophils. Data are presented as mean ± SD. Statistical significance was determined using two‐way ANOVA with Tukey's post hoc test for (a–h), and one‐way ANOVA with Tukey's post hoc test for (i). *p < 0.05, **p < 0.01, ***p < 0.001.

Analysis of temporal changes in immune response to SARS‐CoV‐derived ssRNAs at baseline (0 h), 4 h, 2 days, and 7 days post‐injection of CoV1 and CoV2 ssRNAs, we observed a significant decrease in WBC and lymphocyte counts at 4 h post‐injection in both age groups. Young mice showed rapid recovery, with WBC and lymphocyte counts returning to baseline levels by 2 days post‐injection. In contrast, aged mice exhibited a delayed recovery, with these cell populations gradually returning to baseline levels by day 7 (Figure 3a,b). This age‐dependent recovery pattern was consistently observed across other immune cell populations, including CD11b^+^ cells, NK cells, and T cells (both CD4^+^ and CD8^+^ subsets) (Figure 3c,d,f–h). However, young mice showed no significant changes in B cell populations after exposure to SARS‐CoV ssRNAs. In aged mice, while B cell populations were higher than in young mice initially, exposure to either CoV1 or CoV2 ssRNA led to a marked decrease in B cell populations. The B cell counts did not recover to levels similar to the control group (GA) until 7 days after ssRNA exposure (Figure 3e).

Given the significance of reduced WBC counts and peripheral blood lymphopenia acting as potential early‐stage diagnostic markers for SARS‐CoV‐2 infection, we focused on the 4‐h time point to explore whether there were differences in immune cell changes between surviving (S) and non‐surviving (NS) aged mice, which received either CoV1 or CoV2 ssRNA challenges. Although 4‐h CoV1 and CoV2 ssRNA exposures significantly decreased immune cell populations in both young and aged mice, there was no difference between the survival and non‐surviving groups in aged mice at this time point (Figure S4). These results suggest that acute immune responses alone may not predict survival outcomes.

To investigate whether baseline immune cell counts could serve as critical predictors of survival in aged mice following SARS‐CoV ssRNA exposure, we analyzed hematological parameters at baseline (0 h). Aged mice receiving CoV1 or CoV2 ssRNA injection were categorized into surviving (S) and non‐surviving (NS) groups based on their subsequent survival outcomes. Comparison of various immune cell populations prior to SARS‐CoV ssRNA injection among young mice, old surviving (S), and old non‐surviving (NS) groups revealed striking differences. The old non‐surviving group exhibited significantly higher counts of WBCs, lymphocytes, monocytes, neutrophils, and basophils compared to both young mice and the old surviving group. Notably, these immune cell populations showed no significant differences between young mice and the old surviving group. Eosinophil counts remained consistent across all three groups (Figure 3i). These findings suggest that elevated baseline levels of specific immune cell populations in aged mice may be strongly associated with adverse outcomes in elderly following SARS‐CoV ssRNA challenge.

### 2.4 Age‐Dependent Cytokine Kinetics and Pre‐Existing Inflammatory State Determine Outcomes in SARS‐CoV ssRNA Exposure

Cytokines play a crucial role in orchestrating immune responses to viral infections. Age‐related differences in cytokine dynamics may underline the increased severity of COVID‐19 in older populations. Our study revealed significantly higher baseline levels of pro‐inflammatory cytokines in aged mice compared to young adults (Figure S5), consistent with the concept of “inflammaging.” This chronic low‐grade inflammatory state potentially predisposes aged individuals to dysregulated responses during SARS‐CoV infection (Suárez‐Reyes and Villegas‐Valverde 2021).

**FIGURE 4:**
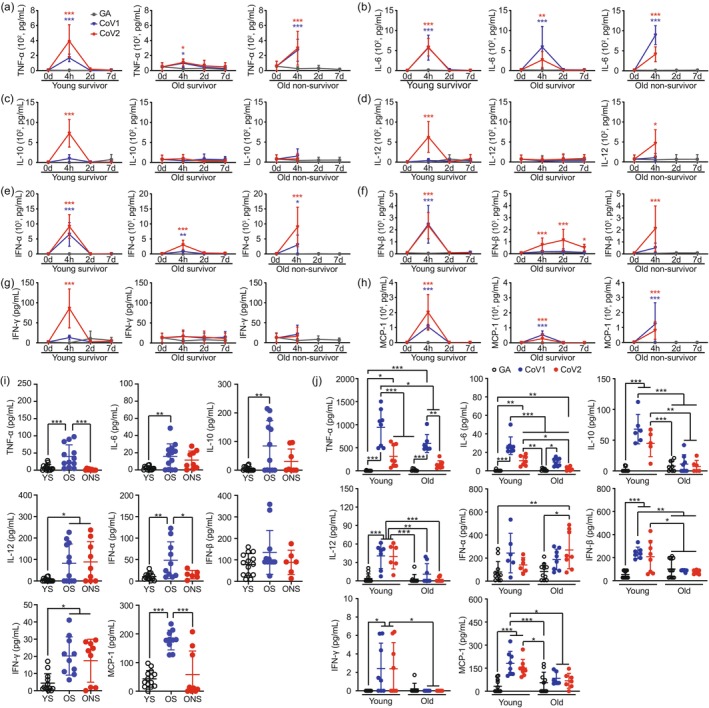
Age‐dependent cytokine dynamics, baseline profiles, and CD11b^+^ cell responses to SARS‐CoV ssRNAs. (a–h) Temporal changes in serum cytokine levels following injection of GA ssRNA (gray), CoV1 ssRNA (blue), or CoV2 ssRNA (red) in young surviving mice, old surviving mice, and old non‐surviving mice. Blood samples were collected at 0 (*n* = 10), 4 h (*n* = 3–7), 2 days (*n* = 3–5), and 7 days (*n* = 3–5) post‐injection. Analyzed cytokines: (a) TNF‐α; (b) IL‐6; (c) IL‐10; (d) IL‐12; (e) IFN‐α; (f) IFN‐β; (g) IFN‐γ; (h) MCP‐1. (i) Baseline (0 h) cytokine levels in young surviving (YS), old surviving (OS), and old non‐surviving (ONS) groups prior to ssRNAs challenge. See also Figure S6 for a consolidated comparison of cytokine responses across different groups at the 4‐h timepoint. (j) Cytokine secretion by CD11b^+^ cells isolated from young and aged mice after 24‐h in vitro exposure to various ssRNAs. Data are presented as mean ± SD. Statistical significance was determined using two‐way ANOVA with Tukey's post hoc test for (a–h), and one‐way ANOVA with Tukey's post hoc test for (i, j); *p < 0.05, **p < 0.01, ***p < 0.001.

Following SARS‐CoV ssRNA exposures, cytokine responses varied markedly across age groups and survival outcomes. Young mice exhibited a controlled and transient increase in cytokine levels, returning to baseline within 2 days. Examination of the cytokine data demonstrates that non‐surviving aged mice displayed pro‐inflammatory cytokine responses (TNF‐α, IFN‐α, and MCP‐1) that were comparable to, and occasionally lower than, those observed in young mice (Figure 4a,e,h). Notably, plasma IL‐6 concentrations rapidly increased in both young and aged mice (including surviving and non‐surviving aged mice) within 4 h of viral ssRNA exposures (Figure 4b). Surviving aged mice exposed to CoV2 ssRNA exhibited a unique pattern characterized by a delayed but sustained elevation in IFN‐β (Figure 4f), a response not observed in other groups.

IL‐10 and IFN‐γ exhibited dramatic induction specifically in young survivor mice with CoV2 ssRNA stimulation (red line), peaking at 4 h post‐stimulation before gradually declining by day 7. In contrast, young mice with CoV1 exposure (blue line) showed minimal cytokine fluctuations, with patterns resembling those observed in both old survivor and old non‐survivor groups regardless of viral exposure type. This suggests a coronavirus type‐specific response in young mice, where only CoV2 elicits robust IL‐10 and IFN‐γ production, while the aged cohorts demonstrate consistently muted responses to both CoV1 and CoV2 stimulation (Figure 4c,g).

IL‐12 levels showed a coronavirus type‐specific response pattern. Young surviving mice with CoV2 exposure demonstrated a significant but transient elevation at 4 h post‐exposure that rapidly returned to baseline by day 2. In contrast, young mice with CoV1 exposure maintained relatively low IL‐12 levels similar to those observed in surviving aged mice with either CoV1 or CoV2 exposure. Interestingly, non‐surviving aged mice exposed to CoV2 ssRNA also showed an IL‐12 elevation at 4 h similar to young survivors with CoV2 exposure, with both groups demonstrating a transient spike that returned to baseline by day 2. This comparable pattern of IL‐12 induction in response to CoV2 contrasts with the minimal response observed in both young mice with CoV1 exposure and surviving aged mice with either viral stimulation (Figure 4d). To facilitate direct comparison of acute‐phase inflammatory responses across all experimental conditions, we provide a consolidated group‐wise analysis of cytokine profiles at the 4‐h timepoint in Figure S6. This comparative visualization highlights the distinct differences in early cytokine responses between CoV1 and CoV2 ssRNA exposures, as well as the age‐dependent and survival‐associated variations in inflammatory mediator production.

Analysis of baseline plasma cytokine concentrations prior to ssRNA injection revealed significant differences among young surviving (YS), old surviving (OS), and old non‐surviving (ONS) mice. TNF‐α, IFN‐α and MCP‐1 concentrations were elevated in OS mice compared to YS mice, while ONS mice exhibited lower baseline levels of these cytokines compared to their surviving counterparts. IL‐6 and IL‐10 concentrations were elevated in the OS mice compared to the YS mice. Both OS and ONS mice showed significantly higher baseline levels of IL‐12 and IFN‐γ compared to the YS group. Baseline IFN‐β levels showed no significant differences among the three groups (Figure 4i). These age‐dependent cytokine profiles revealed distinct immune response patterns to SARS‐CoV ssRNAs characterized by cytokine‐specific variations in kinetics and magnitude rather than a universally heightened inflammatory state in aged non‐survivors.

### 2.5 Aging Selectively Uncouples Cytokine Synthesis and Secretion in CD11b ^+^ Cells Responding to SARS‐CoV ssRNAs

The production and release of cytokines from innate immune cells are critical responses to inflammation and infection (Lacy and Stow 2011). CD11b is an integrin expressed on various innate immune cells, including granulocytes, monocytes, DCs, and NK cells (Ley et al. 2007). Given the age‐associated changes in innate immunity and the central role of CD11b^+^ cells in the early response to viral infections (Franceschi et al. 2018), we investigated the cellular basis underlying the observed age‐dependent plasma cytokine profiles. Thus, CD11b^+^ cells were isolated from the bone marrow of young and aged mice, cultured in vitro, and treated with DOTAP‐encapsulated ssRNA to directly assess their response to SARS‐CoV ssRNA stimulation (Figure S7a). Gene expression analysis revealed that SARS‐CoV ssRNA exposures significantly increased the transcription of multiple cytokines in CD11b^+^ cells from both young and aged mice, with similar magnitudes of induction (Figure S7b–f,i). However, IFN‐β and IFN‐γ expression remained unaltered across age groups and ssRNA treatments (Figure S7g,h), suggesting a selective regulation of cytokine gene expression within CD11b^+^ cells in response to viral stimuli.

Analysis of secreted cytokines in the culture medium revealed significant age‐related differences (Figure 4j). Young CD11b^+^ cells showed a marked increase in the secretion of TNF‐α, IL‐6, IL‐10, IL‐12, IFN‐β, IFN‐γ, and MCP‐1 following exposure to CoV1 and CoV2 ssRNAs. Although IFN‐α also showed an increasing trend, it did not reach statistical significance. In contrast, while aged CD11b^+^ cells exhibited some increase in cytokine secretion upon viral ssRNA stimulation, the overall levels were substantially lower compared to young cells. This discrepancy between gene expression and cytokine secretion in young versus aged CD11b^+^ cells presents a striking contrast to our in vivo observations. This finding reveals the complexity of age‐related modulation of immune responses, suggesting that secretion mechanisms may play a crucial role in shaping the systemic inflammatory response to SARS‐CoV ssRNAs in aged individuals.

### 2.6 Age‐Related Dysregulation of IRF7 Signaling and SNARE‐Mediated Cytokine Trafficking in CD11b ^+^ Cells Responding to SARS‐CoV ssRNAs

**FIGURE 5:**
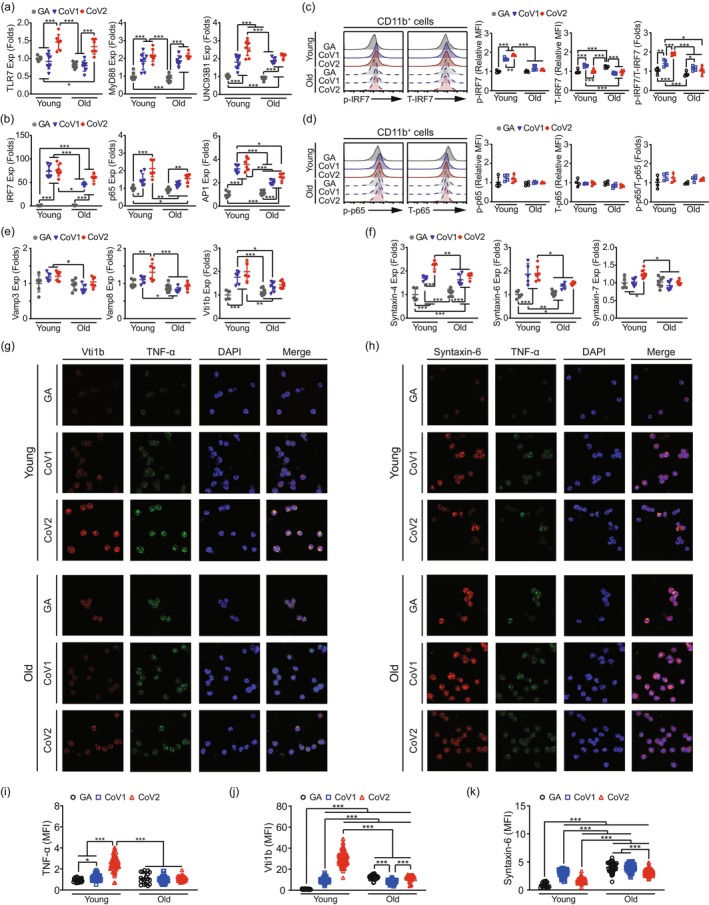
Age‐associated dysregulation of IRF7 signaling and SNARE‐mediated cytokine trafficking in CD11b^+^ cells. (a, b) Relative mRNA expression levels of TLR7 signaling components and downstream signaling molecules in CD11b^+^ cells isolated from young and aged mice after 24‐h exposure to various ssRNAs. (a) Analyzed genes in the TLR7 pathway: TLR7, MyD88, UNC93B1. (b) Analyzed genes downstream of TLR7: IRF7, p65 (NF‐κB subunit), AP1. Flow cytometric analysis of (c) phosphorylated IRF7 (p‐IRF7), total IRF7 (T‐IRF7), and p‐IRF7/T‐IRF7 ratios, as well as (d) phosphorylated p65 (p‐p65), total p65 (T‐p65), and p‐p65/T‐p65 ratios in CD11b^+^ cells from young and aged mice after 24‐h ssRNA treatment. Quantification of mean fluorescence intensity (MFI) of phosphorylated and total proteins were shown. (e, f) Relative mRNA expression levels of SNARE family genes in CD11b^+^ cells isolated from young and aged mice after 24‐h ssRNA exposure. Analyzed genes: (e) Vamp3, Vamp8, Vti1b; (f) Syntaxin‐4, Syntaxin‐6, Syntaxin‐7. (g, h) Immunofluorescence staining of CD11b^+^ cells from young and aged mice treated with GA, CoV1, or CoV2 ssRNAs for 24 h. (g) Co‐staining of Vti1b (red), TNF‐α (green), and DAPI (blue). (h) Co‐staining of Syntaxin‐6 (red), TNF‐α (green), and DAPI (blue). (i–k) Quantification of immunofluorescence staining showing the mean fluorescence intensity (MFI) of: (i) TNF‐α; (j) Vti1b; (k) Syntaxin‐6. Data are presented as mean ± SD. *n* = 5–6 per group. Statistical significance was determined using two‐way ANOVA with Tukey's post hoc test. *p < 0.05, **p < 0.01, ***p < 0.001.

The differential cytokine secretion observed between CD11b^+^ cells from young and aged mice (Figure 4j) prompted us to investigate age‐related changes in innate immune signaling pathways. We examined the TLR7 signaling pathway, which recognizes viral ssRNA, and its downstream effectors in response to SARS‐CoV ssRNA stimulation. In CD11b^+^ cells isolated from bone marrow, both CoV1 and CoV2 ssRNA treatments induced significant upregulation of the TLR7 receptor complex components (TLR7, MyD88, and UNC93B1) in young and aged cells, with no significant age‐dependent differences observed (Figure 5a). However, the expression of key downstream transcription factors showed distinct age‐related patterns. Specifically, CD11b^+^ cells from young mice demonstrated marked increases in IRF7 and AP1 expression following both CoV1 and CoV2 ssRNA exposures, while aged CD11b^+^ cells exhibited significantly attenuated responses compared to the young cells with CoV ssRNA treatments (Figure 5b). The p65 expression pattern showed a similar trend, with enhanced induction in young and aged mice, particularly after CoV2 stimulation (Figure 5b).

Flow cytometric analysis revealed age‐related defects in IRF7 activation in CD11b^+^ cells after CoV ssRNA stimulation. CD11b^+^ cells isolated from young mice showed marked increases in phosphorylated IRF7 (p‐IRF7)/total IRF7 (T‐IRF7) levels after CoV ssRNA stimulation, while aged CD11b^+^ cells exhibited reduced p‐IRF7/T‐IRF7, compared to the young CoV ssRNA‐treated groups (Figure 5c). Regarding p65 phosphorylation, CD11b^+^ cells from either young or aged mice exhibited a similar ratio of phosphorylated p65 to total p65 between GA and viral ssRNA treatment, suggesting that aging does not impair the activation capacity of the p65 pathway in these cells (Figure 5d).

To identify which specific CD11b^+^ cell populations contribute to the observed age‐related defects in IRF7 signaling, we performed immunophenotypical analysis of CD11b^+^ cell subsets. Flow cytometric characterization revealed that granulocytes constituted the predominant population within CD11b^+^ cells in both young (84.78% ± 2.28%) and aged (82.74% ± 1.46%) mice, with monocyte and natural killer (NK) cells showing significant age‐associated expansion (Figure S8a,b).

Analysis of IRF7 activation within specific subpopulations revealed that granulocytes, the predominant CD11b^+^ subset, exhibited significant enhancement of IRF7 activation in young granulocytes following both CoV1 and CoV2 ssRNA stimulation, whereas this response was attenuated in aged counterparts (Figure S8c). Interestingly, monocytes, DCs, and NK cells showed comparable p‐IRF7/T‐IRF7 ratios between young and aged populations following CoV ssRNA stimulation (Figure S8d–f), indicating that the age‐dependent IRF7 signaling defect was primarily confined to granulocytes. These findings provide mechanistic insight into how aging selectively impacts specific immune cell subsets that may interface with age‐dependent cytokine secretion machinery.

Based on our observations of impaired IRF7 activation in aged mouse CD11b^+^ cells (Figure 5c), we further investigated downstream molecular mechanisms that could link defective pattern‐recognition receptor signaling to altered cytokine secretion patterns. Previous research has established that TLR activation in DCs leads to differential expression of SNAREs, which are critical for membrane fusion and cytokine secretion (Collins et al. 2014). Syntaxins, a key SNARE family, have been implicated in aging biology, with Syntaxin‐4 overexpression extending lifespan by 33% while preserving metabolic function in aged mice (Oh et al. 2015). This finding suggests SNARE proteins may serve as molecular links between cytokine secretion, immune function, and longevity regulation.

Examination of SNARE family expression patterns revealed distinct age‐related differences in response to CoV ssRNAs. Young CD11b^+^ cells showed consistent and significant upregulation of Vti1b in response to both CoV1 and CoV2 stimulation, whereas aged CD11b^+^ cells exhibited an attenuated response (Figure 5e, right panel). Additionally, analysis of VAMP family members revealed that Vamp8 expression was modestly elevated in young CD11b^+^ cells following CoV2 stimulation, an effect not observed in aged CD11b^+^ cells (Figure 5e, middle panel), while Vamp3 expression remained unchanged in both young and aged CD11b^+^ cells upon CoV ssRNA stimulation (Figure 5e, left panel). For Syntaxin expression, we observed stimulus‐specific patterns: Syntaxin‐4 was predominantly induced by CoV2 in young CD11b^+^ cells with minimal response in aged cells (Figure 5f, left panel), while Syntaxin‐6 showed significant induction by both CoV1 and CoV2 in young CD11b^+^ cells but remained unresponsive in aged CD11b^+^ cells (Figure 5f, middle panel). Syntaxin‐7 showed modest induction only in young CD11b^+^ cells in response to CoV2 ssRNA treatment (Figure 5f, right panel). These differential SNARE expression patterns suggest that aging might selectively impair distinct components of the vesicle trafficking machinery in response to specific viral RNA motifs, potentially contributing to the altered cytokine secretion profiles observed in aged immune cells.

Immunofluorescence analysis revealed age‐dependent alterations in cytokine trafficking machinery in CD11b^+^ cells. In young cells, 24‐h CoV ssRNA stimulation (both CoV1 and CoV2 ssRNAs) significantly enhanced intracellular TNF‐α expression (Figure 5g–i) compared to GA controls, with CoV2 inducing the strongest response (Figure 5i). Young CD11b^+^ cells demonstrated coordinated upregulation of vesicle trafficking proteins Vti1b (Figure 5g,j) and Syntaxin‐6 (Figure 5h,k) following CoV stimulation, with substantially increased colocalization between TNF‐α and these SNARE proteins (Figure 5g,h). Intriguingly, aged CD11b^+^ cells exhibited significantly elevated baseline expression of both Vti1b and Syntaxin‐6 in GA controls. Despite this elevated baseline, CoV ssRNA stimulation failed to further induce trafficking protein expression or enhance TNF‐α production in aged cells (Figure 5g–k). Critically, colocalization between Vti1b or Syntaxin‐6 and TNF‐α was markedly reduced in aged cells (Figure 5g,h), indicating potential disruption of the spatial coordination necessary for effective cytokine trafficking.

To address which specific CD11b^+^ cell subsets might be responsible for the observed age‐associated alterations in vesicular trafficking machinery, we performed flow cytometric analysis of intracellular Vti1b and Syntaxin‐6 expression across different myeloid cell subpopulations (Figure S9). The age‐dependent pattern of Vti1b expression observed in total CD11b^+^ cells (Figure 5j) was consistently recapitulated across all myeloid subsets examined, including granulocytes, monocytes, DCs, and NK cells. Specifically, all subsets from young mice showed low baseline Vti1b expression that was significantly upregulated following CoV ssRNA stimulation, while cells from aged mice displayed elevated baseline Vti1b levels that remained unresponsive to CoV stimulation (Figure S9a). Though less pronounced statistically, Syntaxin‐6 expression followed similar age‐dependent trends across the different myeloid subpopulations (Figure S9b). These findings suggest that the age‐associated dysregulation in SNARE‐mediated vesicular transport is a widespread phenomenon across multiple myeloid cell subsets, potentially contributing to impaired cytokine secretion in response to coronavirus RNA in aged individuals.

### 2.7 Adoptive Transfer of Young CD11b ^+^ Cells Rejuvenates Age‐Dysregulated Immune Responses to SARS‐CoV ssRNAs

**FIGURE 6:**
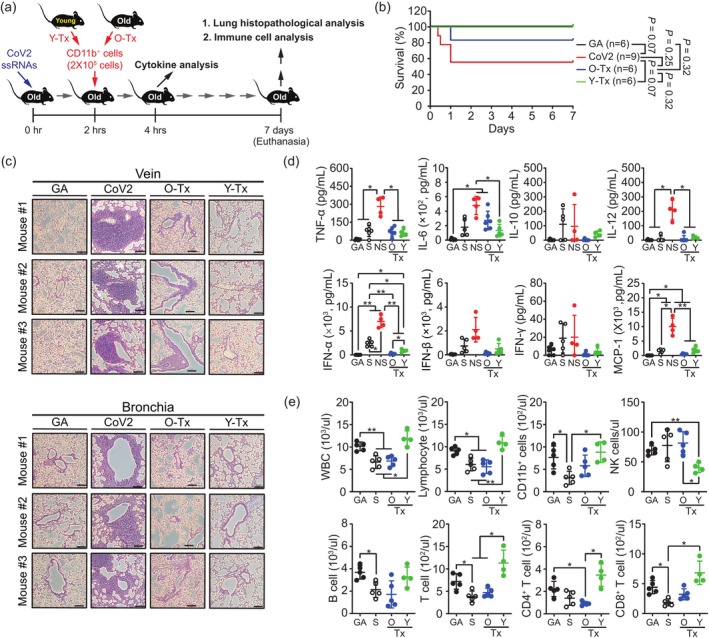
Adoptive transfer of young CD11b^+^ cells improves survival rates and immune function in aged mice exposed to SARS‐CoV‐2 ssRNA. (a) Schematic representation of the experimental design illustrating the adoptive transfer protocol of young (Y‐Tx) and old CD11b^+^ cells (O‐Tx) into aged mice following CoV2 ssRNA challenge. (b) Kaplan–Meier survival analysis comparing aged mice exposed to CoV2 ssRNA with and without the adoptive transfer of CD11b^+^ cells. GA as a control. Statistical significance was determined using the log‐rank Mantel‐Cox test. (c) Representative lung histology (H&E staining) from aged mice and treatment (Tx) groups 7 days post‐ssRNA administration. (d) Changes in plasma cytokine levels in GA, aged survivor (S) and non‐survivor (NS), and Tx groups after 4 h of CoV2 ssRNA exposure. (e) Hematological parameters in treated mice 7 days after CoV2 ssRNA injection. Data are presented as mean ± SD. Statistical significance was determined using one‐way ANOVA with Tukey's post hoc test; *p* < 0.05, **p < 0.01, ***p < 0.001 compared to relevant controls. Scale bar: 100 μm.

Our observations of differential cytokine secretion capabilities between CD11b^+^ cells from young and aged mice led us to investigate whether age‐related immune dysfunction might be amenable to intervention. To test this hypothesis, we examined whether adoptive transfer of CD11b^+^ cells could restore immune responses in aged mice exposed to SARS‐CoV‐2 ssRNA. We isolated CD11b^+^ cells from both young and aged mice, then administered 2 × 10^5^ purified CD11b^+^ cells to each aged recipient mouse 2 h after exposure to CoV2 ssRNA (Figure 6a).

Survival analysis revealed significant differences in mortality rates among treatment groups. Transfer of young CD11b^+^ cells (Y‐Tx) conferred complete protection with 100% survival over the 7‐day observation period. Notably, transfer of aged CD11b^+^ cells (O‐Tx) also provided substantial protection, resulting in 83.33% survival. In contrast, aged mice receiving no cell transfer but exposed to CoV2 ssRNA demonstrated significantly lower survival (55.56%). Control mice receiving GA ssRNA maintained 100% survival throughout the experiment (Figure 6b). These findings indicate that while both young and aged CD11b^+^ cells confer protection, young cells exhibit superior efficacy.

Histopathological examination of lung tissues revealed striking differences between treatment groups (Figure 6c). Aged mice exposed to CoV2 ssRNA without cell transfer exhibited severe inflammatory cell infiltration in both vascular and bronchial regions, with marked disruption of normal lung architecture. In contrast, aged recipients of young CD11b^+^ cells following CoV2 ssRNA exposure demonstrated substantially reduced pulmonary inflammation, with near‐normal alveolar structure preservation comparable to GA ssRNA controls. Aged CD11b^+^ cell transfer recipients exhibited an intermediate phenotype, with moderate inflammatory infiltrates that were nonetheless less severe than untreated CoV2‐exposed mice (Figure 6c).

Analysis of plasma cytokine levels at 4 h post‐ssRNA injection revealed that adoptive transfer effectively modulated the inflammatory cytokine response. Aged non‐surviving mice (NS) exhibited markedly elevated plasma levels of multiple cytokines, particularly TNF‐α, IL‐6, IL‐12, IFN‐α and MCP‐1, compared to GA ssRNA controls. Among aged survivors without cell transfer (S), cytokine elevations were less pronounced but still significant for IFN‐α and MCP‐1. Remarkably, when aged mice received either young (Y‐Tx) or aged (O‐Tx) CD11b^+^ cells 2 h after CoV2 ssRNA exposure, plasma levels of TNF‐α, IFN‐α, IL‐12, and MCP‐1 were largely normalized to levels comparable with GA controls. Interestingly, IL‐6 remained moderately elevated in the O‐Tx group but was effectively normalized in Y‐Tx recipients, suggesting differential regulation of this inflammatory mediator between young and aged CD11b^+^ cells (Figure 6d).

Immunological cell analysis at 7 days post‐ssRNA exposure revealed significant alterations in circulating immune cell populations (Figure 6e). Aged surviving mice without cell transfer (S) showed significantly depleted immune cell counts compared to GA control, including marked reductions in WBCs, lymphocytes, CD11b^+^ cells, B cells, total T cells, and both CD4^+^ and CD8^+^ T cell subsets. Strikingly, aged mice receiving young CD11b^+^ cells (Y‐Tx) demonstrated complete restoration or even enhancement of these immune cell populations to levels equivalent to GA control. Although aged CD11b^+^ cell transfer (O‐Tx) showed intermediate beneficial effects on CD11b^+^ populations, these mice still maintained significantly lower counts of lymphocytes, total T cells, and T cell subsets compared to both GA controls and Y‐Tx recipients (Figure 6e).

These findings demonstrate that adoptive transfer of young CD11b^+^ cells effectively ameliorated SARS‐CoV ssRNA‐induced immune dysfunction in aged mice, restoring survival rates, normalizing cytokine profiles, and preserving immune cell populations. The partial protection conferred by aged CD11b^+^ cells suggests that cellular augmentation provides some benefit, but the superior protective effects of young cells indicate that qualitative aspects of CD11b^+^ cell function are critical determinants of protection. Although these findings suggest the potential of cell‐based approaches for addressing age‐related immune dysfunction during viral challenges, further research would be necessary to determine whether similar strategies might be applicable in clinical settings.

## 3 Discussion

The COVID‐19 pandemic has underscored the critical importance of understanding age‐related immune dysfunction. Our study, utilizing a novel SARS‐CoV‐2‐derived ssRNA mouse model, provides crucial insights into the age‐dependent immunological responses that mirror clinical observations in human COVID‐19 patients. This model effectively recapitulates key aspects of SARS‐CoV‐2 infection while circumventing biosafety and ethical constraints associated with live virus studies (Choudhary et al. 2022). In addition, these age‐dependent immune profiles demonstrated that our animal model effectively recapitulates key aspects of human COVID‐19 pathogenesis (Bartleson et al. 2021).

Our study demonstrates that aging profoundly impacts the immune response to SARS‐CoV ssRNAs through multiple mechanisms, affecting both cellular immunity and cytokine dynamics, with direct implications for survival outcomes in aged mice (Table 2). Following viral ssRNA exposure, aged mice developed rapid lymphopenia, a hallmark of severe COVID‐19 in humans (Xing et al. 2023). Critically, non‐surviving aged mice displayed significantly higher baseline white blood cell, lymphocyte, monocyte, neutrophil, and basophil counts compared to both aged survivors and young mice (Table 2), mirroring the hyperinflammatory state observed in severe COVID‐19 cases among the elderly (Xing et al. 2023). Notably, aged mice exhibited elevated baseline levels of pro‐inflammatory cytokines, including TNF‐α, IL‐6, and MCP‐1, consistent with the “inflammaging” phenomenon observed in older adults (Bartleson et al. 2021). This pre‐existing inflammatory state may predispose the elderly to dysregulated responses upon viral challenge. The cytokine profiles in our model closely parallel those reported in human studies. Non‐surviving aged mice exhibited exaggerated early increases in pro‐inflammatory cytokines, particularly TNF‐α, IFN‐α, and MCP‐1 (Table 2), mirroring the cytokine storm phenomenon associated with severe SARS‐CoV infection in elderly patients. In contrast, surviving aged mice displayed a distinct profile (Table 2). Notably, these survivors also demonstrated a unique pattern of delayed yet sustained IFN‐β elevation following CoV ssRNA challenge (Figure 4f). This distinctive IFN‐β response suggests a potential protective role, aligning with recent observations in human COVID‐19 cases (Chien et al. 2006; Del Valle et al. 2020; Zhang et al. 2004).

**TABLE 2:** Summary of age‐dependent inflammatory responses and mortality in response to SARS‐CoV ssRNAs challenge from our mouse model.

Items	Pre‐existing inflammatory status			Early post‐infection (4‐h) inflammatory status		
	Young mice	Aged mice		Young mice	Aged mice	
		Surviving	Non‐surviving		Surviving	Non‐surviving
Immune cell profiles						
WBC	+	+	++	NS		
Lymphocyte	+	+	++			
Monocyte	+	+	+++			
Neutrophil	+	+	+++			
Basophil	+	+	++			
Cytokine profiles						
TNF‐α	+	+++++	+	+++	+	+++
IFN‐α	+	+++++	+	+++++++	+	+++++
MCP‐1	+	+++	+	+++	+	+++

Abbreviation: NS, no significant difference.

Our study employed multiple complementary approaches to elucidate the complexity of aging‐related cytokine dynamics, identifying CD11b^+^ cells as central mediators of the dysregulated immune response observed in aged mice (Graphical abstract). CD11b^+^ cells, which include granulocytes, monocytes, DCs, and NK cells, play a critical role in pathogen recognition, antigen presentation, cytokine regulation, etc. With aging, these cells experience functional decline, including impaired type I interferon (IFN) responses and dysregulated cytokine secretions, which disrupts immune homeostasis and exacerbates inflammaging, leaving older individuals more vulnerable to infections (Ajoolabady et al. 2024). Our immunophenotypic analysis revealed that granulocytes exhibit the most pronounced IRF7 signaling defects with aging. This finding is particularly significant as neutrophils, the predominant granulocyte population, undergo substantial functional alterations with aging. Aged neutrophils are characterized by diminished phagocytosis, reduced migration ability, and impaired oxidative burst, while simultaneously demonstrating enhanced neutrophil extracellular trap (NET) formation and increased mitochondrial ROS production (Ling and Xu 2024). The selective impairment of IRF7 signaling in granulocytes we observed likely represents a mechanistic link between neutrophil aging and the dysregulated cytokine responses in elderly individuals with severe SARS‐CoV infections.

Additionally, we identified significant impairment in SNARE‐mediated cytokine secretion within aged CD11b^+^ cells for the first time in this study. SNARE proteins, particularly Vti1b and Syntaxin‐4/6, play crucial roles in vesicle trafficking and fusion, facilitating the precise and regulated release of cytokines (Murray and Stow 2014; Stow et al. 2006). Although SARS‐CoV ssRNA stimulation robustly upregulated these SNARE components in young mice, enabling efficient cytokine secretion, CD11b^+^ cells from aged mice showed markedly reduced expression of SNARE proteins and diminished colocalization of TNF‐α with Vti1b and Syntaxin‐6. This SNARE dysfunction in CD11b^+^ cells is particularly significant as these cells must rapidly produce and secrete large quantities of cytokines during viral infection, making them exceptionally dependent on efficient vesicular trafficking machinery. The functional consequences of this defect were evident in our ex vivo cytokine secretion assays, where CD11b^+^ cells from aged mice exhibited markedly altered cytokine release patterns despite comparable transcriptional responses to viral stimuli. These findings suggest that post‐transcriptional mechanisms, particularly those involved in protein trafficking and secretion, represent a previously underappreciated aspect of immune aging that may be especially relevant in rapid‐response innate immune populations like CD11b^+^ cells.

The dual impairment of both IRF7 signaling and SNARE‐mediated cytokine secretion mechanisms creates a compound deficit in the aged immune response, potentially explaining the increased susceptibility to severe outcomes in older individuals infected with SARS‐CoV. These mechanistic insights illuminate the multifaceted nature of immune aging and suggest several potential therapeutic strategies for addressing age‐related immune dysfunction. Previous studies have demonstrated that age‐related immune dysfunction can be modulated through blood exchange between young and old animals. Rebo et al. showed that a single heterochronic blood exchange between young and old mice led to rapid changes in multiple tissues, with notably different effects depending on the tissue type examined. Although old blood exerted dominant inhibitory effects on young brain neurogenesis and cognitive performance, young blood showed significant beneficial effects on aged muscle regeneration and liver function. These findings established that age‐specific systemic factors can rapidly influence tissue regenerative capacity without requiring prolonged exposure or shared organ systems (Rebo et al. 2016). Our adoptive transfer experiments extend these observations to viral immunity, demonstrating that transplantation of young CD11b^+^ cells into aged mice restored normal cytokine profiles, reduced inflammatory lung pathology, and significantly improved survival outcomes following SARS‐CoV ssRNA challenge. Importantly, while Rebo et al. focused on tissue regeneration after injury, our work reveals that cellular‐based immune rejuvenation can effectively restore protective antiviral responses in aged animals. The superior protective effects of young CD11b^+^ cells compared to aged CD11b^+^ cells further suggest that qualitative aspects of immune cell function, rather than merely increased cell numbers, are critical determinants of protection against coronavirus challenges in aged individuals.

The isolation of CD11b^+^ cells offers distinct technical advantages, including the ability to collect multiple innate immune cell populations within a single procedure, potentially streamlining the manufacturing process compared to therapies requiring the isolation of specific cell types. This comprehensive approach significantly reduces the complexities associated with current cellular therapies. Unlike CAR‐T cell therapies that require extensive ex vivo manipulation, which can alter cellular metabolism, migration capabilities, and cytoskeletal organization (Sudarsanam et al. 2022), CD11b^+^ cells maintain their native functional properties with minimal manipulation. Although CAR‐T cell manufacturing requires 7–14 days of ex vivo expansion with costly cytokine supplementation and genetic modifications (Sudarsanam et al. 2022), our CD11b^+^ isolation protocol achieves therapeutically relevant cell numbers within a significantly shorter timeframe.

Furthermore, previous reports address the viral escape mechanisms that limit B cell‐based therapies, which often struggle when confronting highly mutable viral antigens with single or few mutations in epitopes (White 2021). CD11b^+^ cells offer broad pattern‐recognition capabilities that are not dependent on specific antigen configurations. Moreover, CD11b^+^ cells circumvent the limitations that constrain NK cell therapy efficacy, which, despite showing promise in early viral clearance, often face challenges with immunopathology and cytokine storm complications (Market et al. 2020). Our approach harnesses cells that naturally migrate to infection sites and coordinate multifaceted innate immune responses without the need for artificial homing receptor engineering that other cellular therapies require for effective tissue targeting. Although our findings underscore the potential of CD11b^+^ cell therapy to overcome these significant challenges in current cellular immunotherapies, we recognize that further research is needed to determine whether these theoretical benefits can translate into practical clinical applications.

## 4 Materials and Methods

The experimental procedures are described in the Supporting Information.

## Author Contributions

Yuan‐I Chang conceptualized the study, designed the experiments, interpreted the data, acquired funding, supervised the project, and contributed to writing – review and editing. Yu‐Xuan Wu conducted in vivo experiments and, in collaboration with Jui‐Yu Chang, managed molecular analyses and cytokine measurements. Chih‐Wei Hu, Chuen‐Mi Yang, and Hsuan‐Ying Lu performed experiments on SARS‐CoV‐2 BA.5‐infected mice. Eric Chang‐Yi Lin developed the flow cytometry panel and gating strategy. Yu‐Xuan Wu, Shuoh‐Wen Chen, and Min‐Hui Chen executed cell fluorescence staining and operated the flow cytometry equipment. Additionally, Yu‐Xuan Wu, Jui‐Yu Chang, and Ching‐Jung Teng carried out in vitro experiments. Ting‐An Lin contributed to data interpretation and provided clinical insights. The manuscript was written by Yuan‐I Chang and Yu‐Xuan Wu with contributions from all authors.

## Conflicts of Interest

The authors declare no conflicts of interest.

## Supporting information


Appendix S1

## Data Availability

The data supporting the findings of this study are available from the corresponding author upon reasonable request. Access to the data will be provided in accordance with institutional policies and ethical guidelines.
