# Correction to “Impaired Cytokine Secretion Contributes to Age‐Dependent Immune Dysfunction in SARS Coronavirus Response and Is Restored by Young CD11b‐Positive Cell Transfer”

**DOI:** 10.1111/acel.70744

**Published:** 2026-09-25

Wu, Y.‐X. , C.‐W. Hu , J.‐Y. Chang , et al. (2025), Impaired Cytokine Secretion Contributes to Age‐Dependent Immune Dysfunction in SARS Coronavirus Response and Is Restored by Young CD11b‐Positive Cell Transfer. Aging Cell, 24: e70154. 10.1111/acel.70154.PMC1241983940583123

In Figure 6c, the lung section shown for Mouse #3 in the CoV2 column (Vein) is the same field as the section shown in Figure 2b (Old, CoV2); it appears in Figure 6c as a crop of that field presented at a larger size. Both panels show an aged mouse 7 days after CoV2 ssRNA administration, stained with hematoxylin and eosin. The re‐use of this image was not declared in the figure's legend.

**FIGURE 6:**
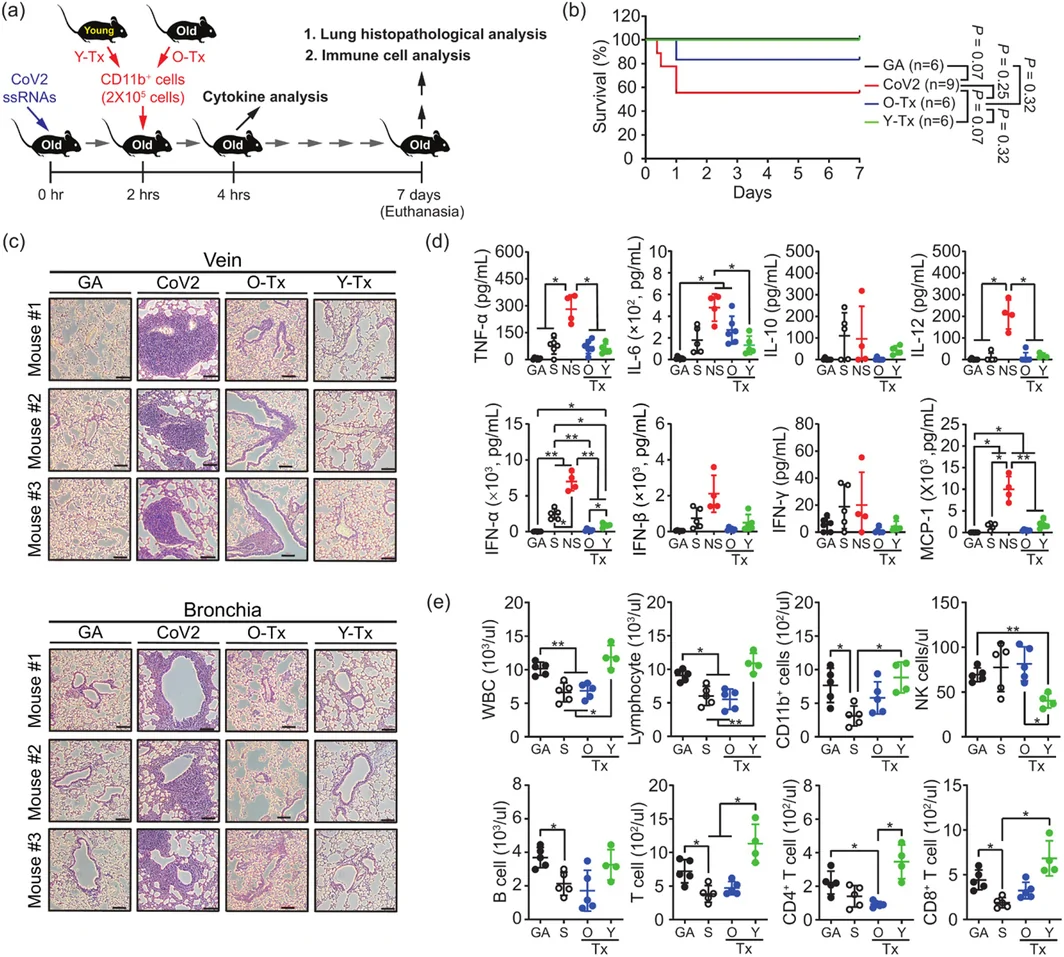
Adoptive transfer of young CD11b^+^ cells improves survival rates and immune function in aged mice exposed to SARS‐CoV‐2 ssRNA. (a) Schematic representation of the experimental design illustrating the adoptive transfer protocol of young (Y‐Tx) and old CD11b^+^ cells (O‐Tx) into aged mice following CoV2 ssRNA challenge. (b) Kaplan–Meier survival analysis comparing aged mice exposed to CoV2 ssRNA with and without the adoptive transfer of CD11b^+^ cells. GA as a control. Statistical significance was determined using the log‐rank Mantel‐Cox test. (c) Representative lung histology (H&E staining) from aged mice and treatment (Tx) groups 7 days post‐ssRNA administration. The section shown for Mouse #3 in the CoV2 column (Vein) is the same field as that shown in Figure 2b (Old, CoV2), reproduced here as a crop presented at a larger size. (d) Changes in plasma cytokine levels in GA, aged survivor (S) and non‐survivor (NS), and Tx groups after 4 h of CoV2 ssRNA exposure. (e) Hematological parameters in treated mice 7 days after CoV2 ssRNA injection. Data are presented as mean ± SD. Statistical significance was determined using one‐way ANOVA with Tukey's post hoc test; *p < 0.05, **p < 0.01, ***p < 0.001 compared to relevant controls. Scale bar: 100 μm.

Accordingly, in the legend to Figure 6, the text “(c) Representative lung histology (H&E staining) from aged mice and treatment (Tx) groups 7 days post‐ssRNA administration.” was incomplete.

This should have read as: “(c) Representative lung histology (H&E staining) from aged mice and treatment (Tx) groups 7 days post‐ssRNA administration. The section shown for Mouse #3 in the CoV2 column (Vein) is the same field as that shown in Figure 2b (Old, CoV2), reproduced here as a crop presented at a larger size.”

Figure 6 along with its corrected legend is provided below. The image itself is unchanged, and the results, interpretation and conclusions of the article are unaffected.

In addition, in the legend to Figure 6 the significance markers were printed as “*p* < 0.05, **p < 0.01, ***p < 0.001”; this should have read as “*p < 0.05, **p < 0.01, ***p < 0.001”. The corrected legend below incorporates both changes.

We apologize for these errors.

