# Supplementary material for: Impaired Cytokine Secretion Contributes to Age‐Dependent Immune Dysfunction in SARS Coronavirus Response and Is Restored by Young CD11b‐Positive Cell Transfer
Source: Aging Cell. 2025 Jun 29;24(9):e70154. doi: 10.1111/acel.70154 (PMC12419839; doi:10.1111/acel.70154)
Supplement: Supplementary file 1 — Appendix S1 [file ACEL-24-e70154-s001.docx]

**Supplemental Information**

**Supplemental Materials and Methods**

**1. 1 Single-strand RNA (ssRNA) Design**

SARS-CoV-1 GU-rich ssRNA (5'-GUCUGAGUGUGUUCUUG-3'; CoV1), based on a previously reported sequence ([Li et al., 2013](#_ENREF_19)), and SARS-CoV-2 ssRNA (5'-GUCAGAGUGUGUACUUG-3'; CoV2) were synthesized by IDT, Inc., USA. Two control ssRNAs were used: a GA-rich ssRNA, derived from CoV1-GU by replacing uracil (U) residues with adenine (A) as described previously ([Li et al., 2013](#_ENREF_19)), and HIV-derived ssRNA40 (InvivoGen, Inc., USA).

**1.2 Mouse Models and *In Vivo* Experiments**

Young (8-12 weeks) and aged (18-24 months) male C57BL/6J mice were obtained from the Laboratory Animal Center of National Yang Ming Chiao Tung University (Taipei Yang Ming Campus, Taiwan). For SARS-CoV-2 infection study, B6.129S2(Cg)-*Ace2^tm1(ACE2)Dwnt^*/J mice were acquired from Jackson Laboratory (Bar Harbor, ME, USA) and provided by Dr. Hu. Mice were housed in specific-pathogen-free conditions with a 12-hour light/dark cycle and ad libitum access to food and water.

C57BL/6J mice received retro-orbital injections of ssRNA (1 mg/kg body weight) complexed with DOTAP (Sigma-Aldrich) in HBS (2:1 ratio, 300 µL total volume). Control mice received DOTAP/HBS mixture without ssRNAs. For survival and histopathological studies, mice were monitored for 7 days post-injection.

For SARS-CoV-2 infection experiments, male B6.129S2(Cg)-*Ace2^tm1(ACE2)Dwnt^*/J mice were intranasally inoculated with 5×10^4^ plaque-forming units (PFU) of SARS-CoV-2 Omicron BA.5 variant in a biosafety level 3 laboratory at the Experimental Animal Center, Institute of Preventive Medicine, National Defense Medical College, Taiwan.

All animal procedures were approved by the Institutional Animal Care and Use Committee (IACUC) of National Yang Ming Chiao Tung University (IACUC number: 1130472, for C57BL/6J mice) and the National Defense Medical Center (IACUC number: AN-112-43, for B6.129S2(Cg)-*Ace2^tm1(ACE2)Dwnt^*/J mice), in accordance with the Guide for the Care and Use of Laboratory Animals.

**1.3 Blood Sampling Procedures**

Blood samples were collected serially from a single cohort of mice to minimize inter-subject variability. Sampling was performed via the retro-orbital sinus under isoflurane anesthesia to reduce stress and discomfort. Strict adherence to humane procedures ensured that the minimum volume of blood necessary for the experiment was drawn to minimize the physiological impact on the animals.

**1.4 CD11b^+^ Cell Isolation and Adoptive Cell Transfer**

For *in vitro* experiments, CD11b^+^ cells were isolated from bone marrow and peripheral blood of young and aged mice using APC-conjugated anti-CD11b antibodies and APC Magnetic Particles (BD Biosciences), following the manufacturer's protocol. Isolated CD11b^+^ cells (2×10^5^ cells/well) were treated with ssRNA/DOTAP complexes for 24 hours.

To investigate the potential rejuvenating effects of CD11b^+^ cells, we performed adoptive cell transfer experiments. Specifically, CD11b^+^ cells (2×10⁵) were isolated from the peripheral blood of young and aged mice using a negative selection strategy to minimize activation effects. This process involved the depletion of B and T cell populations using biotin-conjugated CD19 and CD90.2 antibodies, followed by separation with Streptavidin Magnetic Beads (BD Biosciences). The purified cells were injected into the aged mice's retro-orbital venous sinus two hours after ssRNA/DOTAP complex treatment. This approach allowed us to assess the impact of young and aged immune cells on ssRNA-induced responses in an aged immune environment.

**1.5 Flow Cytometry Analysis**

Peripheral blood cells were stained with antibodies against CD45 (30-F11), CD90.2 (Thy-1.2) (53-2.1), CD4 (RM4-5), CD8 (53-6.7), CD19 (1D3), CD11b (M1/70), NK1.1 (PK136), Ly6G(1A8), and Ly6c (AL-21) (all from BD Biosciences). Analysis was performed using a Beckman Coulter Cytoflex S system.

**1.6 Cytokine Measurements**

Plasma cytokine levels were quantified using the cytometric bead array (CBA) mouse inflammation kit (BD Biosciences) and the LEGENDplex™ multiple analyte flow assay kit (BioLegend). All procedures were performed according to the manufacturers' instructions. Both assays were analyzed using a CytoFLEX S flow cytometer (Beckman Coulter). The following cytokines were measured: TNF-α, IL-6, IL-10, IL-12, IFN-α, IFN-β, IFN-γ, MCP-1.

**1.7 Gene Expression Analysis**

Total RNA was isolated from CD11b^+^ cells using the RNeasy Plus Mini Kit (Qiagen) and reverse transcribed using the RevertAid First-Strand cDNA Synthesis Kit (Thermo Fisher Scientific). Quantitative PCR was performed using Fast SYBR™ Green Master Mix on a QuantStudio 3 Real-Time PCR System (Applied Biosystems). Relative gene expression levels were calculated using the 2^-ΔΔCt^ method, with GAPDH as the internal control. Primer sequences are listed in Table S1. All samples were run in triplicate, and fold changes were calculated relative to the control group (young mice treated with GA ssRNA).

**1.8 Phospho-flow Analysis**

Activation of intracellular signaling molecules was evaluated by phospho-flow cytometry according to previously established protocols ([Kong et al., 2016](#_ENREF_15)). In brief, CD11b^+^ cells were stimulated with ssRNA/DOTAP complexes, subsequently fixed, permeabilized, and immunolabeled with phospho-specific antibodies targeting phosphorylated IRF7 and NF-κB p65, alongside antibodies recognizing total IRF7 and NF-κB p65 proteins (BD Biosciences). Multiparametric flow cytometric analysis was conducted using a Beckman Coulter Cytoflex S platform.

**1.9 Immunofluorescence Staining**

CD11b^+^ cells isolated from young and aged mice were treated with ssRNA/DOTAP complexes for 24 hours. Cells were spun down on coverslips, then fixed with 4% paraformaldehyde, permeabilized with 0.1% Triton X-100, and incubated with primary antibodies against Vti1b, Syntaxin-6, and TNF-α (BD Biosciences) overnight at 4°C. After washing, cells were incubated with appropriate fluorophore-conjugated secondary antibodies for 1 hour at room temperature. Nuclei were counterstained with DAPI. Images were acquired using a Zeiss LSM880 confocal microscope with an oil immersion objective (Carl Zeiss AG, Germany). Image analysis was performed using ZEN software (Carl Zeiss AG, Germany).

**1.10 Statistical analysis**

Data were analyzed using GraphPad Prism version 6 or 10 (GraphPad, Inc., USA). The unpaired two-tailed Student's *t*-tests, as well as one-way ANOVA or two-way ANOVA with Tukey's post-hoc tests, were used as appropriate. Results are presented as mean ± SD, with *P* < 0.05 considered statistically significant.

**1.11 Language Editing with AI-based Tools**

To enhance clarity and improve English usage, portions of the manuscript text were refined using large language model (LLM)-based tools, including ChatGPT (OpenAI, USA) and Claude (Anthropic, USA). These tools were employed solely for language editing and were not involved in the generation, interpretation, or analysis of experimental data. All AI-assisted content was carefully reviewed and revised by the authors to ensure accuracy, scientific validity, and compliance with journal policies.

**Table S1. The qPCR primer list**

| Genes | Forward primer | Reverse primer |
| --- | --- | --- |
| TNF-α | 5’-GGTGCCTATGTCTCAGCCTCTT-3’ | 5’-GCCATAGAACTGATGAGAGGGAG-3’ |
| IL-6 | 5’-GAGGATACCACTCCCAACAGACC-3’ | 5’-AAGTGCATCATCGTTGTTCATACA-3’ |
| IL-10 | 5’-CGGGAAGACAATAACTGCACCC-3’ | 5’-CGGTTAGCAGTATGTTGTCCAGC-3’ |
| IL-12 | 5’-ACGAGAGTTGCCTGGCTACTAG-3’ | 5’-CCTCATAGATGCTACCAAGGCAC-3’ |
| IFN-α | 5’-GGATGTGACCTTCCTCAGACTC-3’ | 5’-ACCTTCTCCTGCGGGAATCCAA-3’ |
| IFN-β | 5’-GCCTTTGCCATCCAAGAGATGC-3’ | 5’-ACACTGTCTGCTGGTGGAGTTC-3’ |
| IFN-γ | 5’-CAGCAACAGCAAGGCGAAAAAGG-3’ | 5’-TTTCCGCTTCCTGAGGCTGGAT-3’ |
| MCP-1 | 5’-GCTACAAGAGGATCACCAGCAG-3’ | 5’-GTCTGGACCCATTCCTTCTTGG-3’ |
| Vamp3 | 5’-AGACCAGAAGCTCTCGGAGCTA-3’ | 5’-ACCAGGACACTGATCCCTATCG-3’ |
| Vamp8 | 5’-CCAGAATGTGGAGCGGATCTTG-3’ | 5’-CCACCTTCTGGGACGTTGTCTT-3’ |
| Vti1b  Syntaxin-4  Syntaxin-6  Syntaxin-7  TLR7  MyD88  UNC93B1 | 5’-AGGAGACCTGAAGTATGGCACG-3’  5’-GTCCCAGCAATTTGTCGAGCTC-3’  5’-TGGAATGCTGGAGTGGCAGATC-3’  5’-GAAGCCAATGTAGAAAGTGCGGA-3’  5’-GTGATGCTGTGTGGTTTGTCTGG-3’  5’-ACCTGTGTCTGGTCCATTGCCA-3’  5’-CTACAGTGGCTTTGAGGTGCTC-3’ | 5’-TGTGGCAATCCGATGAGAACGC-3’  5’-TCCTCGTCAGACACCATTCCAG-3’  5’-CCAACTGCTCATCCTGCTGTTC-3’  5’-TCACGATTCCGACCACGAGGAT-3’  5’-CCTTTGTGTGCTCCTGGACCTA-3’  5’-GCTGAGTGCAAACTTGGTCTGG-3’  5’-GCTATGAGCAGGTATGCCAGTC-3’ |

**
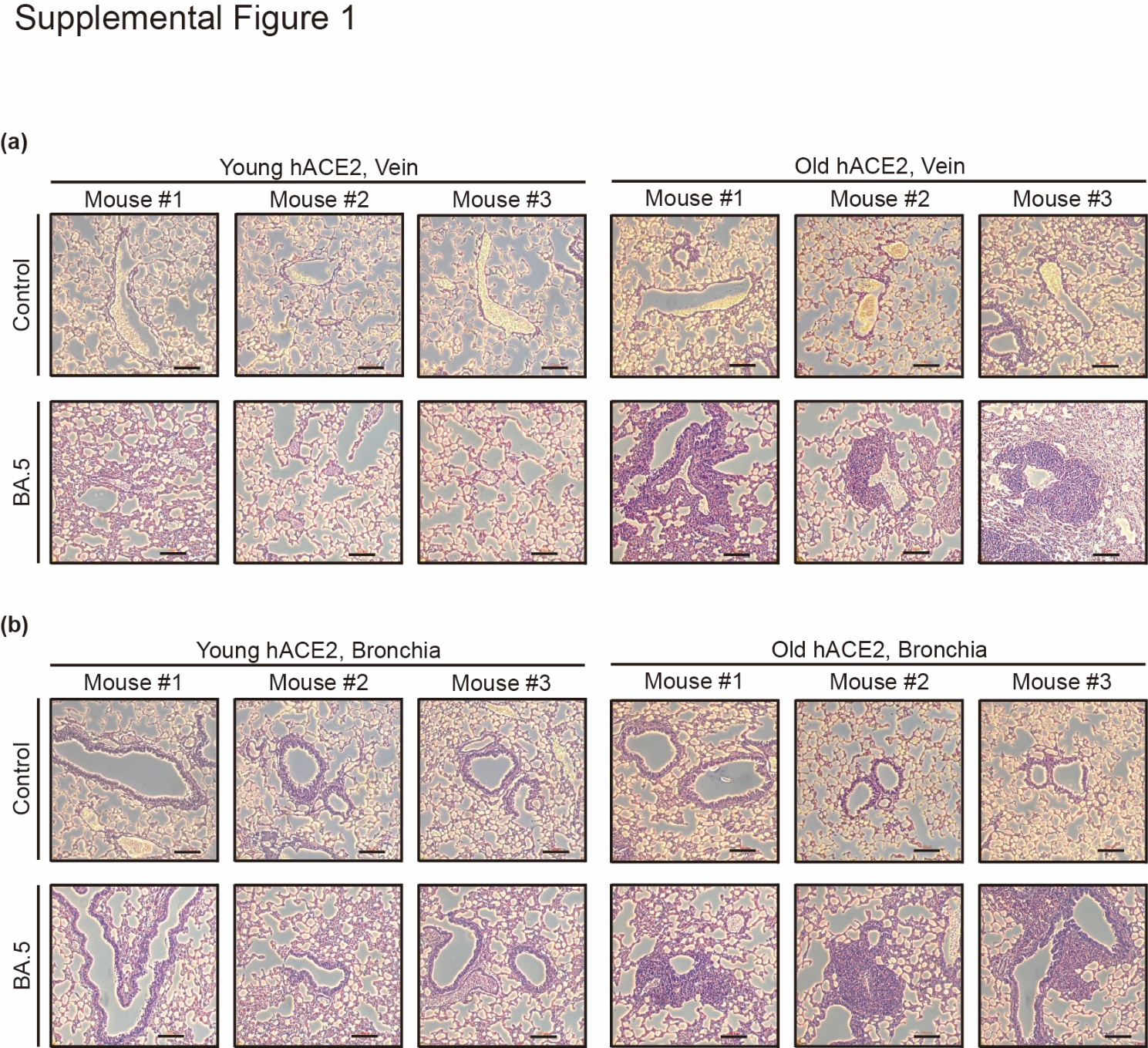
**

**Figure S1. Histopathological analysis of lung sections in young and aged mice after SARS-CoV-2 BA.5 infection.**

Representative hematoxylin and eosin (H&E)-stained lung sections comparing (a) veins and (b) bronchial areas between young and aged mice at 7 days post-injection with either live SARS-CoV-2 BA.5 variant or control. Scale bar: 100 μm.


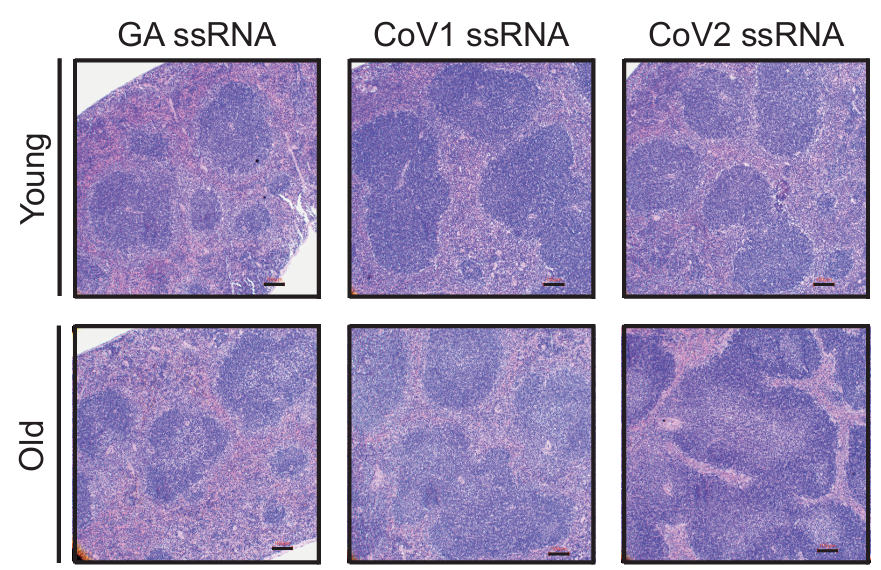


**Figure S2. Histopathological evaluation of spleen sections from young and aged mice following injection with SARS-CoV-derived ssRNAs.**

Representative hematoxylin and eosin (H&E) stained spleen sections from young and aged mice 7 days after retro-orbital injection of GA, CoV1-GU, CoV2-GU ssRNAs. Scale bar: 100 μm.

**
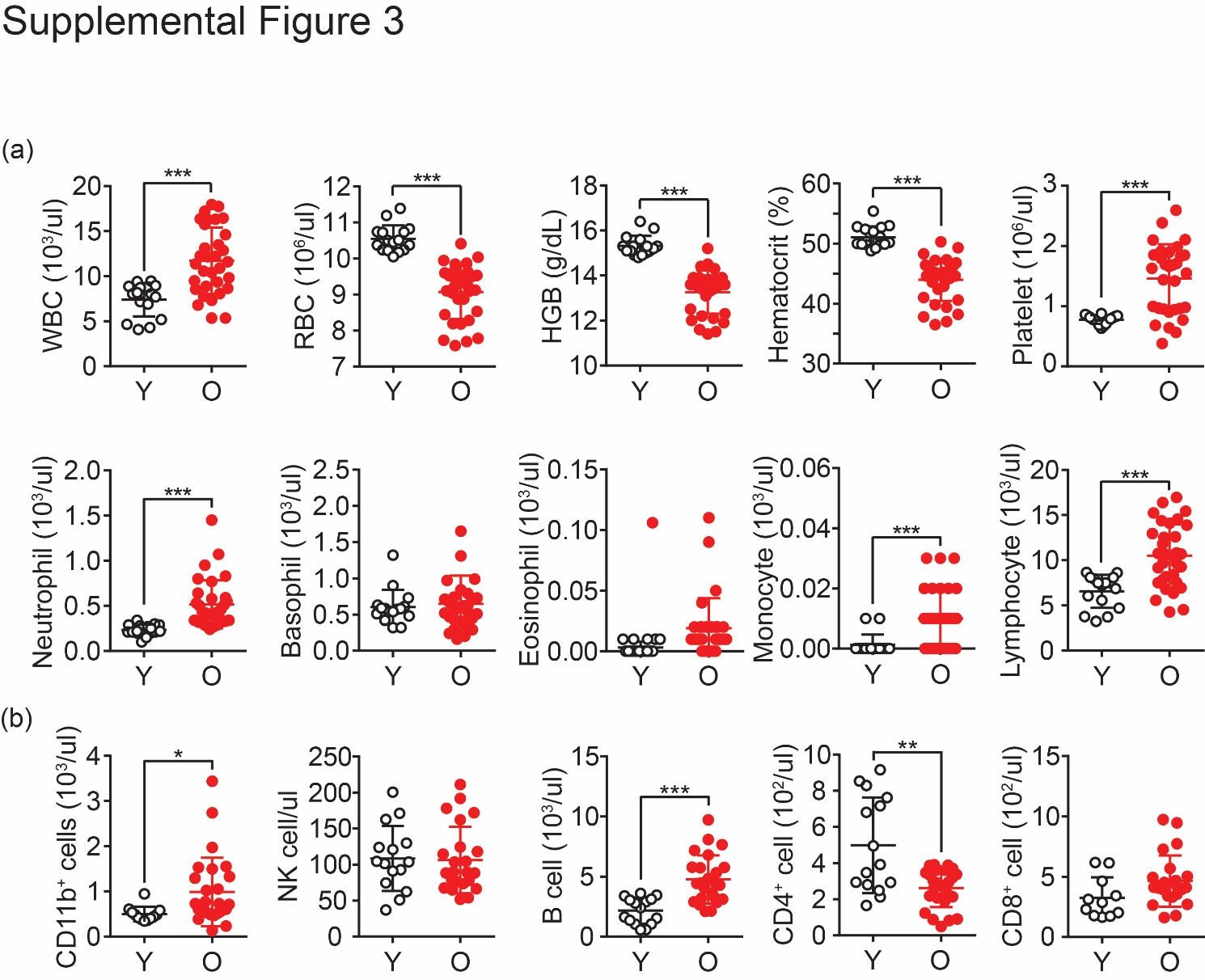
**

**Figure S3. Baseline immune cell profiles in young and aged mice.**

(a) Comparison of complete blood count (CBC) parameters between young and aged mice, including white blood cells (WBC), red blood cells (RBC), hemoglobin (HGB), hematocrit, platelets, neutrophils, basophils, eosinophils, monocytes, and lymphocytes. (b) Flow cytometric quantification of immune cell subpopulations, including CD11b^+^ cells, natural killer (NK) cells, B cells, CD4^+^ T cells, and CD8^+^ T cells, in young and aged mice. Data are presented as mean ± SD. Statistical significance was determined using unpaired two-tailed Student's *t*-tests; **P* < 0.05, ***P* < 0.01, ****P* < 0.001.


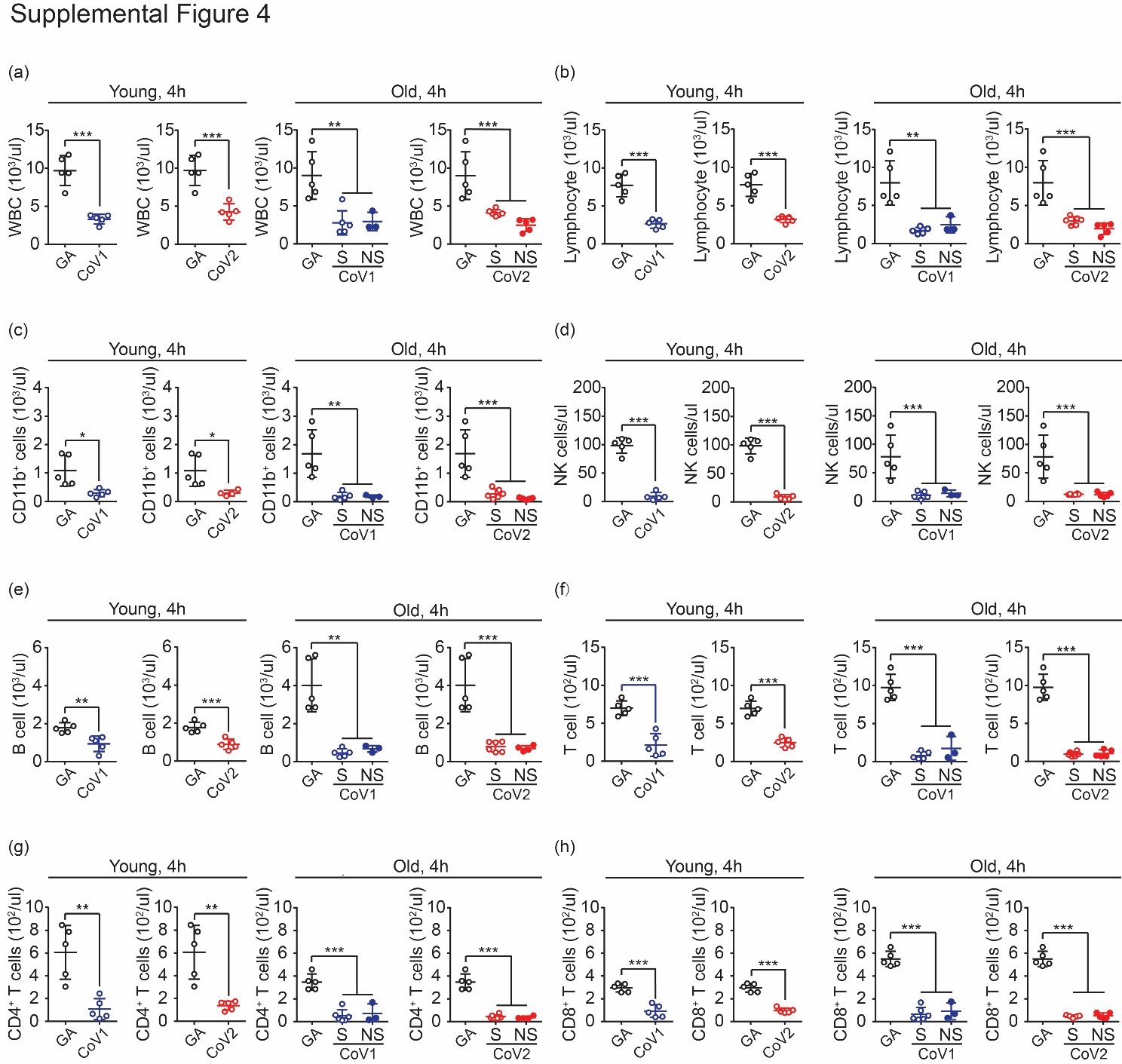


**Figure S4. Early immune cell responses to ssRNA challenge in young and aged mice with survival outcome analysis.**

Analysis of immune cell populations in young and aged mice 4 hours after GA, CoV1-GU, or CoV2-GU ssRNA administration. Aged mice were classified as survivors (S) or non-survivors (NS). Analyzed parameters: (a) White blood cells (WBC); (b) Lymphocytes; (c) CD11b^+^ cells; (d) Natural killer (NK) cells; (e) B cells; (f) Total T cells; (g) CD4^+^ T cells; (h) CD8^+^ T cells. Data are presented as mean ± SD. Statistical significance was determined using unpaired two-tailed Student's *t*-tests or one-way ANOVA with Tukey's post-hoc test; **P* < 0.05, ***P* < 0.01, ****P* < 0.001.


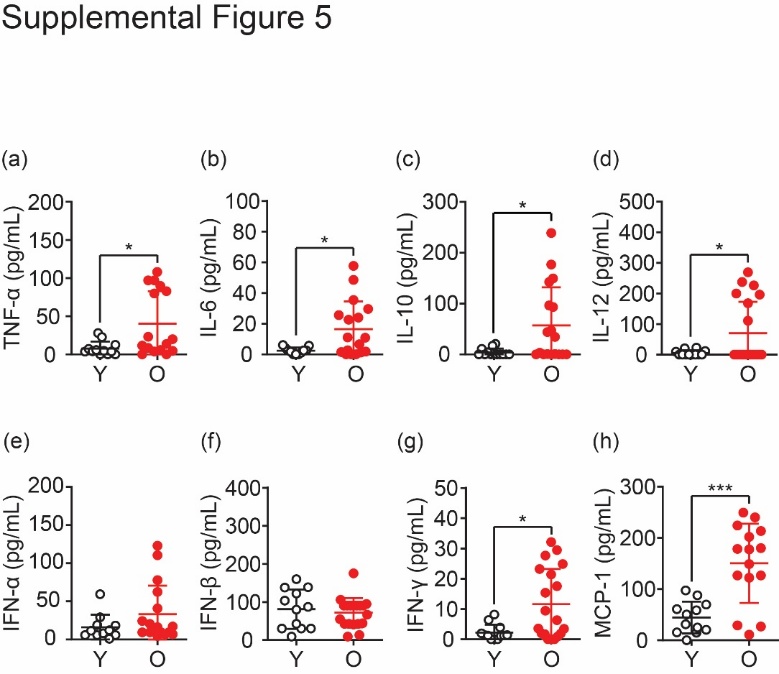


**Figure S5. Baseline plasma cytokine profiles in young and aged mice.**

Comparison of plasma cytokine concentrations between young and aged mice under basal conditions. Analyzed cytokines: (a) TNF-α; (b) IL-6; (c) IL-10; (d) IL-12; (e) IFN-α; (f) IFN-β; (g) IFN-γ; (h) MCP-1. Data are presented as mean ± SD. Statistical significance was determined using unpaired two-tailed Student's *t*-tests; **P* < 0.05, ****P* < 0.001.


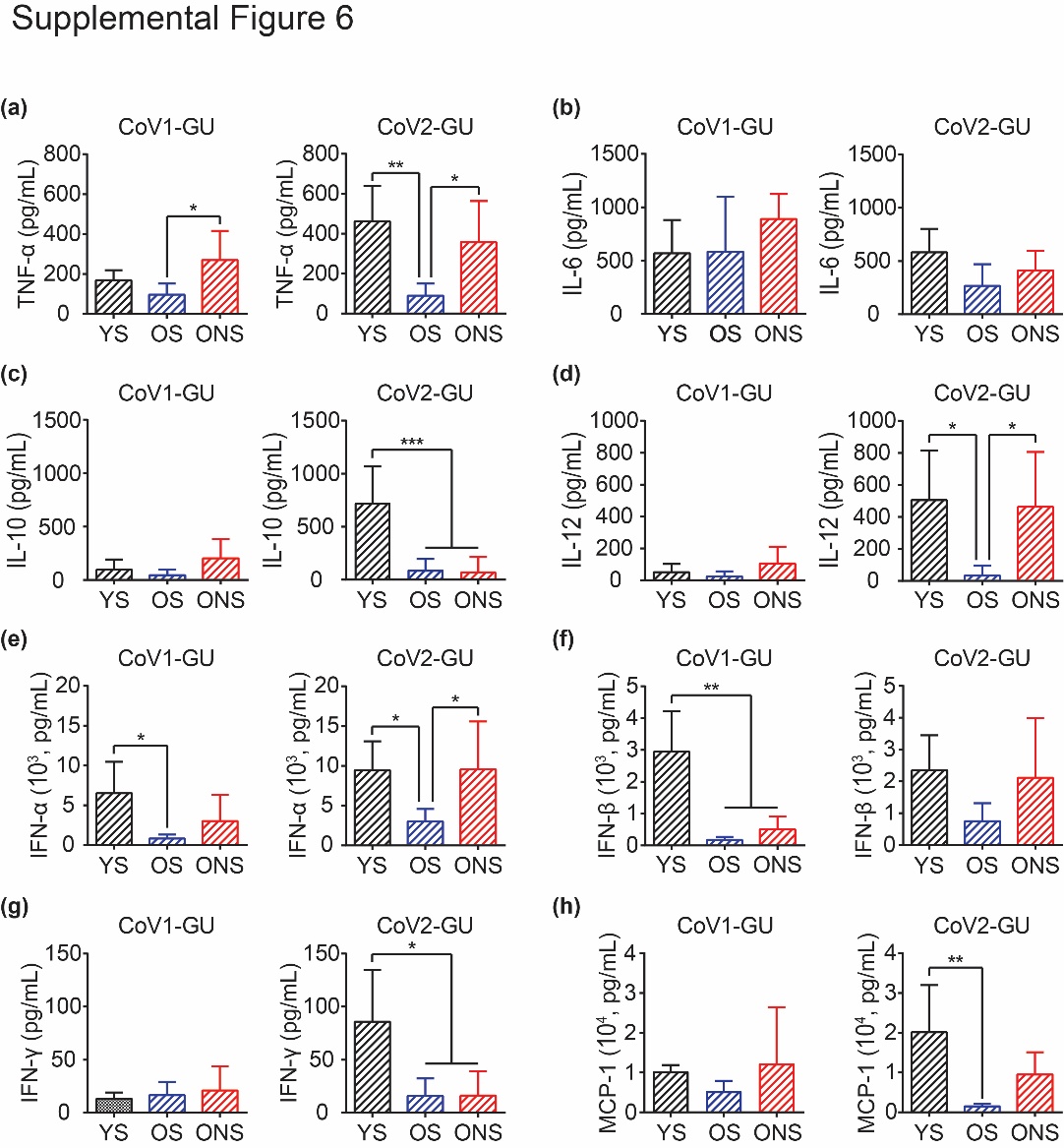


**Figure S6. The cytokine responses across different groups at 4 hours post-ssRNA injection.**

Cytokine levels were measured in young surviving (YS), old surviving (OS), and old non-surviving (ONS) mice 4 hours after retro-orbital administration of CoV1-GU or CoV2-GU ssRNAs. Analyzed cytokines include: (a) TNF-α; (b) IL-6; (c) IL-10; (d) IL-12; (e) IFN-α; (f) IFN-β; (g) IFN-γ; (h) MCP-1. Data are presented as mean ± SD. Statistical significance was determined using one-way ANOVA with Tukey's post-hoc test; **P* < 0.05, ***P* < 0.01, ****P* < 0.001.


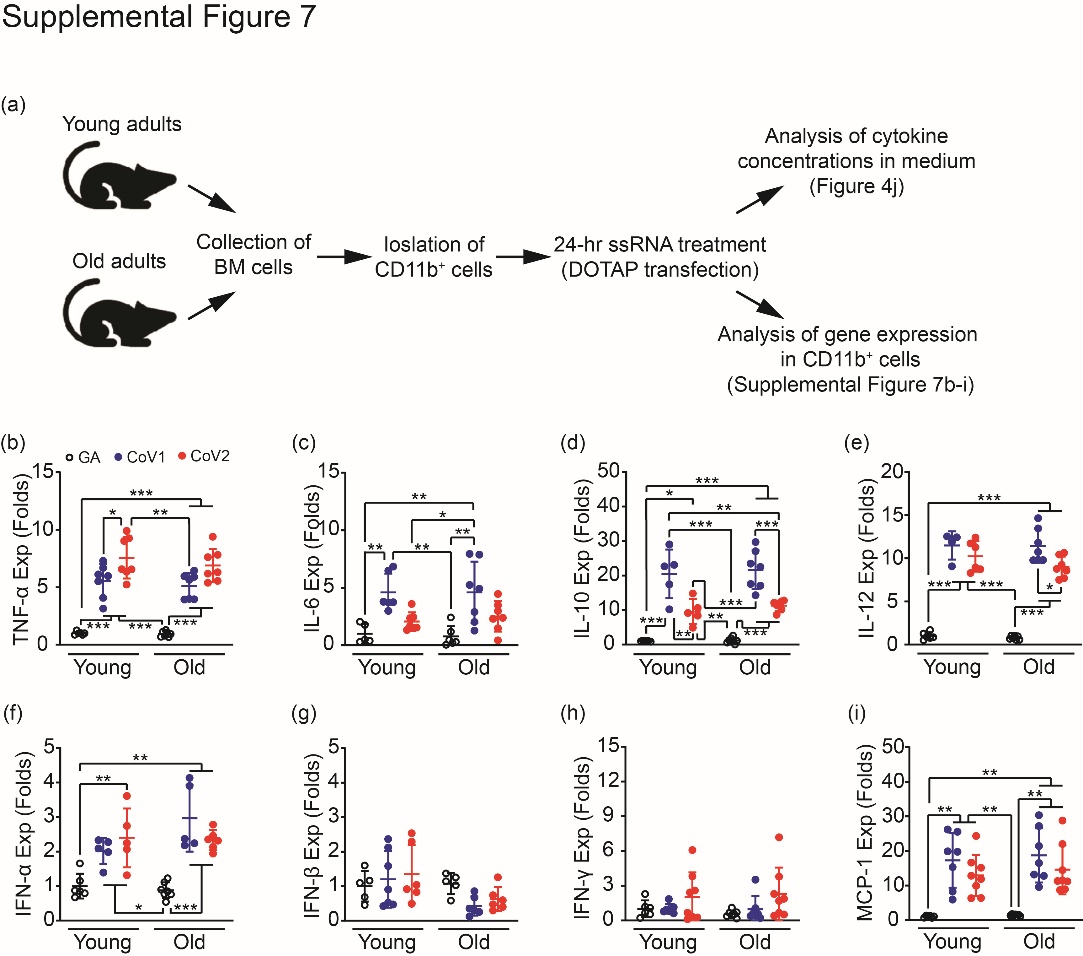


**Figure S7. Cytokine gene expression in CD11b^+^ cells following SARS-CoV ssRNAs challenge.**

(a) Experimental design schematic for *in vitro* CD11b^+^ cell stimulation and analysis. (b-i) Relative mRNA expression levels of cytokines in CD11b^+^ cells isolated from young and aged mice after 24-hour exposure to GA, CoV1-GU, or CoV2-GU ssRNAs. Analyzed cytokines: (b) TNF-α; (c) IL-6; (d) IL-10; (e) IL-12; (f) IFN-α; (g) IFN-β; (h) IFN-γ; (i) MCP-1. Data are presented as mean ± SD. Statistical significance was determined using two-way ANOVA with Tukey's post-hoc test; **P* < 0.05, ***P* < 0.01, ****P* < 0.001.

**
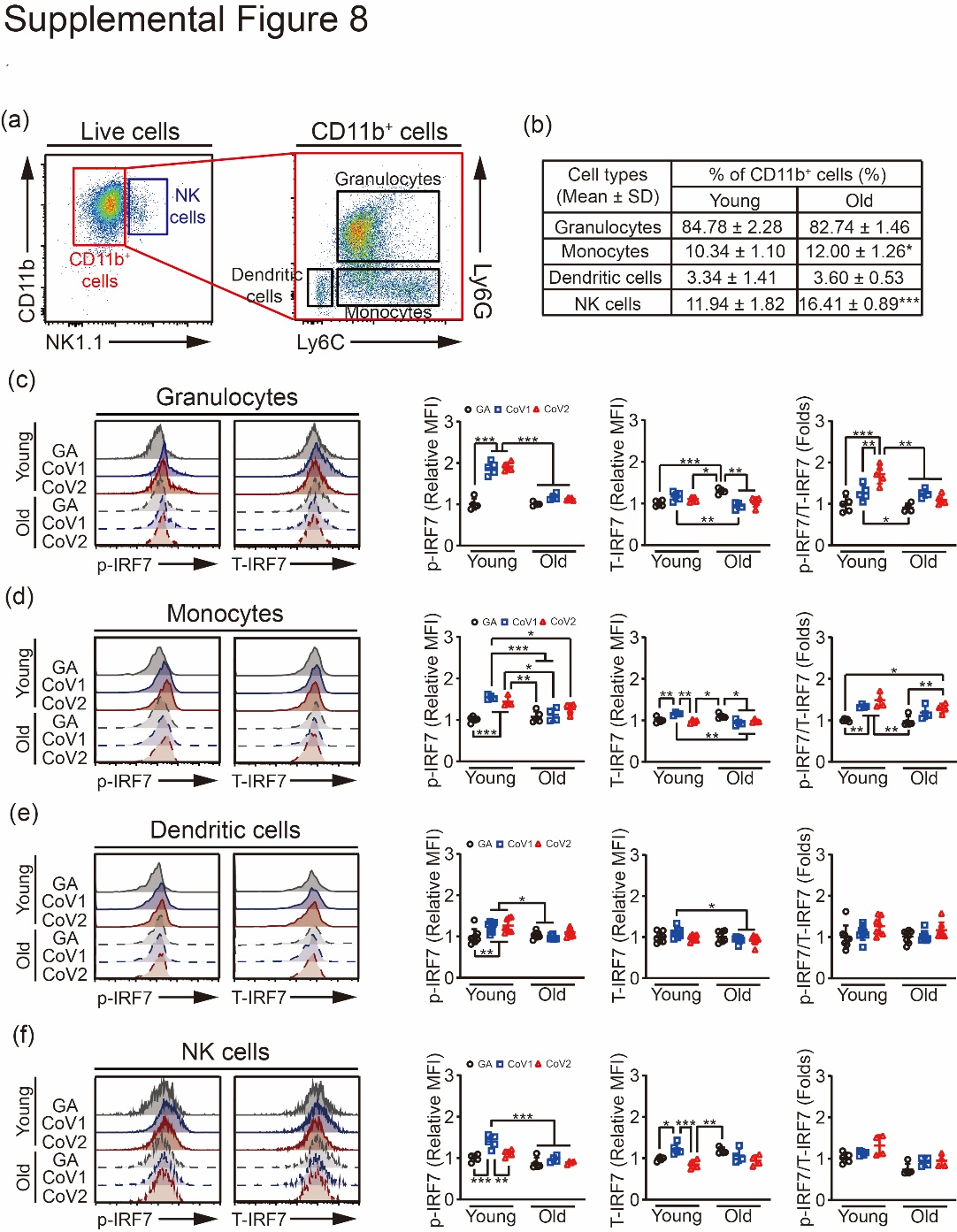
**

**Figure S8. Flow cytometric analysis of CD11b^+^ subsets and subset-specific IRF7 activation following SARS-CoV ssRNA stimulation in young and aged cells.**

(a) Flow cytometric gating strategy illustrating the identification of CD11b^+^ cell subsets. (b) The distribution of CD11b^+^ cell subsets in young and old mice. (c–f) Relative mean fluorescence intensity (MFI) levels of phosphorylated IRF7 (p-IRF7) and total IRF7 (T-IRF7) in CD11b^+^ subsets isolated from young and aged mice after 24-hour stimulation with GA, CoV1-GU, or CoV2-GU ssRNA. Analyzed subsets include (c) granulocytes, (d) monocytes, (e) dendritic cells (DCs), and (f) natural killer (NK) cells. Data are presented as mean ± SD. Statistical significance was determined using two-way ANOVA with Tukey's post-hoc test; **P* < 0.05, ***P* < 0.01, ****P* < 0.001.


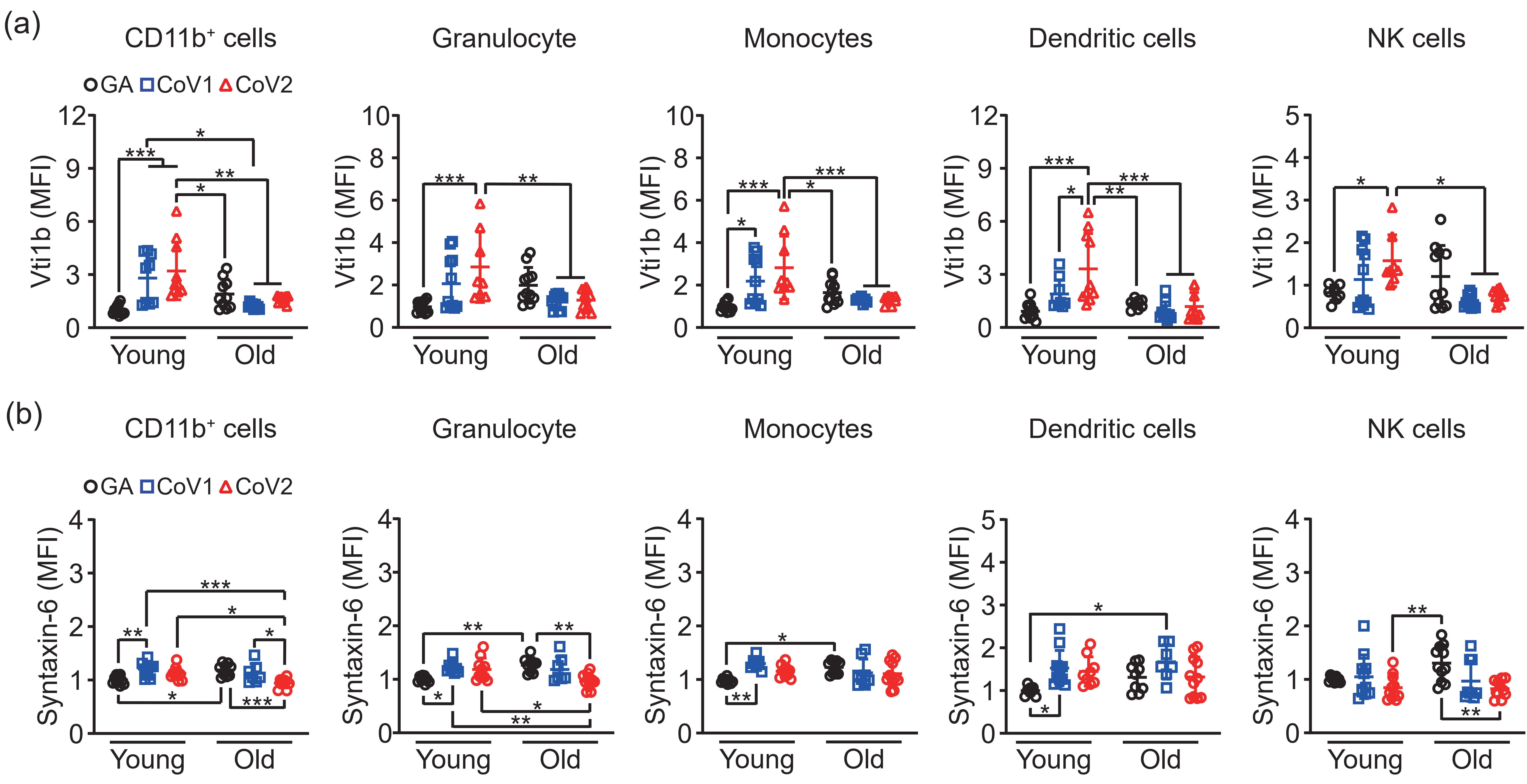


**Figure S9. Expression of Vti1b and Syntaxin-6 in CD11b^+^ subsets from young and aged mice following SARS-CoV ssRNA stimulation.**

The flow cytometric analysis reveals the relative mean fluorescence intensity (MFI) of (a) Vti1b and (b) Syntaxin-6 in total CD11b^+^ cells and subsets, including granulocytes, monocytes, dendritic cells, and natural killer (NK) cells, isolated from young and aged mice after 24-hour stimulation with GA, CoV1-GU, or CoV2-GU ssRNAs. Data are presented as mean ± SD. Statistical significance was determined by two-way ANOVA with Tukey's post-hoc test; **P* < 0.05, ***P* < 0.01, ****P* < 0.001.
